# Comparative Live-Cell Imaging Analyses of SPA-2, BUD-6 and BNI-1 in *Neurospora crassa* Reveal Novel Features of the Filamentous Fungal Polarisome

**DOI:** 10.1371/journal.pone.0030372

**Published:** 2012-01-24

**Authors:** Alexander Lichius, Mario E. Yáñez-Gutiérrez, Nick D. Read, Ernestina Castro-Longoria

**Affiliations:** 1 Department of Microbiology, Center for Scientific Research and Higher Education of Ensenada (CICESE), Ensenada, Baja California, Mexico; 2 Fungal Cell Biology Group, Institute of Cell Biology, Rutherford Building, The University of Edinburgh, Edinburgh, United Kingdom; University of Aberdeen, United Kingdom

## Abstract

A key multiprotein complex involved in regulating the actin cytoskeleton and secretory machinery required for polarized growth in fungi, is the polarisome. Recognized core constituents in budding yeast are the proteins Spa2, Pea2, Aip3/Bud6, and the key effector Bni1. Multicellular fungi display a more complex polarized morphogenesis than yeasts, suggesting that the filamentous fungal polarisome might fulfill additional functions. In this study, we compared the subcellular organization and dynamics of the putative polarisome components BUD-6 and BNI-1 with those of the *bona fide* polarisome marker SPA-2 at various developmental stages of *Neurospora crassa*. All three proteins exhibited a yeast-like polarisome configuration during polarized germ tube growth, cell fusion, septal pore plugging and tip repolarization. However, the localization patterns of all three proteins showed spatiotemporally distinct characteristics during the establishment of new polar axes, septum formation and cytokinesis, and maintained hyphal tip growth. Most notably, in vegetative hyphal tips BUD-6 accumulated as a subapical cloud excluded from the Spitzenkörper (Spk), whereas BNI-1 and SPA-2 partially colocalized with the Spk and the tip apex. Novel roles during septal plugging and cytokinesis, connected to the reinitiation of tip growth upon physical injury and conidial maturation, were identified for BUD-6 and BNI-1, respectively. Phenotypic analyses of gene deletion mutants revealed additional functions for BUD-6 and BNI-1 in cell fusion regulation, and the maintenance of Spk integrity. Considered together, our findings reveal novel polarisome-independent functions of BUD-6 and BNI-1 in *Neurospora*, but also suggest that all three proteins cooperate at plugged septal pores, and their complex arrangement within the apical dome of mature hypha might represent a novel aspect of filamentous fungal polarisome architecture.

## Introduction

Cell polarity regulation is a central process during cell morphogenesis. The establishment and maintenance of cell polarity occurs in response to diverse external and internal signals, which control processes such as cell symmetry breaking, polarized tip growth, and cellular compartmentalization, as well as intracellular transport of RNA, proteins and organelles.

Many key aspects of the molecular basis of cell polarity regulation have been elucidated using yeast models, including *Saccharomyces cerevisiae*, *Schizosaccharomyces pombe*, and *Candida albicans* (reviewed for example in [1], [2], [3], [4], [5], [6], [7]), but certain facets of filamentous fungal morphogenesis are more complex and cannot be explained by the yeast paradigm [8], [9], [10]. These most notably include: (1) the ability to simultaneously establish several axes of polarized growth from the individual spore thereby giving rise to functionally distinct cell protrusions (e.g. germ tubes and conidial anastomosis tubes [CATs]) [11], [12], [13], (2) the ability to permanently maintain polarized tip growth and form tubular hyphae which can achieve much higher tip growth rates than can yeasts [14], [15], and (3) to establish interconnected germling and hyphal networks by cell fusion [16]. Some of the molecular components conserved between yeasts and filamentous fungi appear to be used in different morphogenetic contexts during filamentous fungal development, and proteins no longer encoded in the yeast genome are additional key features responsible for the more complex, multicellular morphology of filamentous fungi.

The tip growth apparatus of vegetative hyphae consists of three major components: the Spitzenkörper (Spk), the polarisome and the exocyst [17], [18], [19]. Together, they contain more than 40 different proteins [20] which, in interaction with the three cytoskeletal polymers F-actin, microtubules and septins, regulate hyphal morphogenesis and tip growth [21], [22]. Targeted secretion of plasma membrane and cell wall components through the exocyst drives tip extension, and is coupled to compensatory endocytosis within a subapical collar [23], [24], [25], rich in F-actin patches [26], [27]. The newly emerging ‘Apical Recycling Model’ accounts for the need to balance exocytosis and endocytosis at the hyphal tip in order to control growth and cell shape, maintain high tip extension rates and recover key plasma membrane proteins (e.g. receptors) back to the growing apex [28]. Therefore, recycling endocytosis can be considered a fourth key component of the hyphal tip growth apparatus.

The polarisome is involved in the establishment, maintenance and termination of polarized cell growth. Proteins known to constitute this complex in budding yeast include the three core components Spa2, Pea2 and Aip3/Bud6, as well as the formin Bni1 [29], [30], [31]. Localization and activation of Bni1 at the cell cortex requires the presence of all three core proteins [31], [32], [33], [34], which together localize in an apical cap driving the directed extension of the bud, mating projection or pseudohypha. All are equally required to delocalize apical actin and terminate mating projection growth in budding yeast [35].

Spa2 is considered to be the central polarisome scaffolding protein that physically interacts with all other components through specific binding domains [29], [36], [37]. Pea2 contains a predicted coiled-coil domain suggesting a possible function in targeted vesicle delivery; its precise molecular role, however, remains obscure. Nevertheless, it has been shown to display interdependent localization with Spa2, and to be required for bipolarization and mating cell fusion [38], [39]. The actin-interacting protein Aip3/Bud6 was initially identified as a protein that besides its association with actin also contains domains which suggested binding to Spa2, Pea2 and Bni1 [29], [40], [41]. The formin Bni1 is stimulated by Bud6 in a positive feedback loop and together they reinforce the assembly of robust actin cables from the cell cortex during budding and mating projection formation, and contractile actomyosin ring formation during cytokinesis [32], [40], [41]. More recent data suggested an additional function of Bud6 in microtubule plus-end capture at the cell cortex, with contributions of formins [42]. Localized assembly of these polarity regulators in the polarisome is maintained through a positive feedback loop from the Cdc42/Cdc24/Bem1 module whose components shuttle between the cytoplasm and plasma membrane [43], [44]. Due to its vital importance in cell polarity regulation, this Cdc42 GTPase module can be considered a fifth core component of the tip growth apparatus.

Homologs of Spa2, Aip3/Bud6 and Bni1 have been identified in a number of other filamentous growing fungi, and the majority already successfully localized in at least one of those species (Table 1). A homolog of Pea2 has so far only been identified in the filamentous yeast, *Ashbya gossypii* [45]. Recent work in *Neurospora crassa* has demonstrated how deletion or loss-of-function of CDC42, RAC-1 or CDC24 lead to severe defects in apical polarity and consequently hyphal morphologies [46], thereby demonstrating the vital role of this GTPase module in filamentous fungal cell polarity regulation. Although many new insights into the inner workings of the polarisome have been gained over the past decades, it is very likely that additional components localizing to this structure will be identified in the near future, revealing further details of its functional differences between yeasts and filamentous fungi.

**Table 1:** Polarisome components in yeast and filamentous fungi.

*S. cerecisiae*	Filamentous fungi	Localization	Species (Reference)
**Spa2**	AgSpa2p	Apex and Septa	*A. gosypii* [45]
	Spa2	Apex	*C. albicans* [66]
	SpaA	Apex	*A. nidulans* [65]
	Spa2	Apex	*U. maydis* [93]
	SpaA	Apex	*A. niger* [94]
	SPA-2	Apex	*N. crassa* [59]
**Pea2** *	(AGR135Cp)	?	*A. gosypii* [18]
**Bni1**	AgBni1	Apex	*A. gossypii* [87]
	Bni1	Apex	*C. albicans* [66], [86]
	SepA	Apex and Septa	*A. nidulans* [74]
	SepA	Apex	*A. niger* [95]
	BNI-1	Apex	*N. crassa* [62]
**Aip3/Bud6**	AgBud6	Apex	*A. gossypii* [18]
	Bud6	Apex	*C. albicans* [66]
	BudA	Septa	*A. nidulans* [65]

*apart from *A. gosypii,* Pea2 homologues have so far not been identified in the genomes of any other filamentous fungal species.

In this study we set out to analyze the subcellular organization and dynamics of SPA-2, BUD-6 and BNI-1 in a wide range of developmental stages of *Neurospora crassa*, in order to characterize the filamentous fungal polarisome more comprehensively, and identify potential differences to other fungal species. Our analysis showed that during early, unicellular developmental stages the filamentous fungal polarisome closely resembles the apical cap configuration known from yeasts, but during later, multicellular developmental stages the three polarisome components SPA-2, BUD-6 and BNI-1 become spatiotemporally separated within the apical dome, and thus adopt a so far unknown polarisome architecture. Furthermore, novel polarisome-independent functions of BUD-6 and BNI-1 have been identified, including the maintenance of Spitzenkörper integrity, cell fusion, septum formation and cytokinesis.

## Results

### Heterologous expression of fluorescently labeled BUD-6

Expression of fluorescently labeled BUD-6 occurred under control of the glucose-repressible *ccg-1* promoter from an ectopically integrated plasmid. However, we did not find evidence that the lack of native expression levels interfered with normal cell biology and colony development in *Neurospora crassa* (Figure S2), suggesting that the constructs were functional and the observed localization patterns reflect the dynamics of the native protein. Due to the absence of conidia, transformation of the Δ*bud-6* gene deletion mutant with *bud-6-gfp* in order to demonstrate phenotypic rescue, was not possible. Therefore, functionality of the fusion protein has not yet been formally proven.

### BUD-6 functioned during polarized tip growth maintenance rather than its establishment

**Figure 1:**
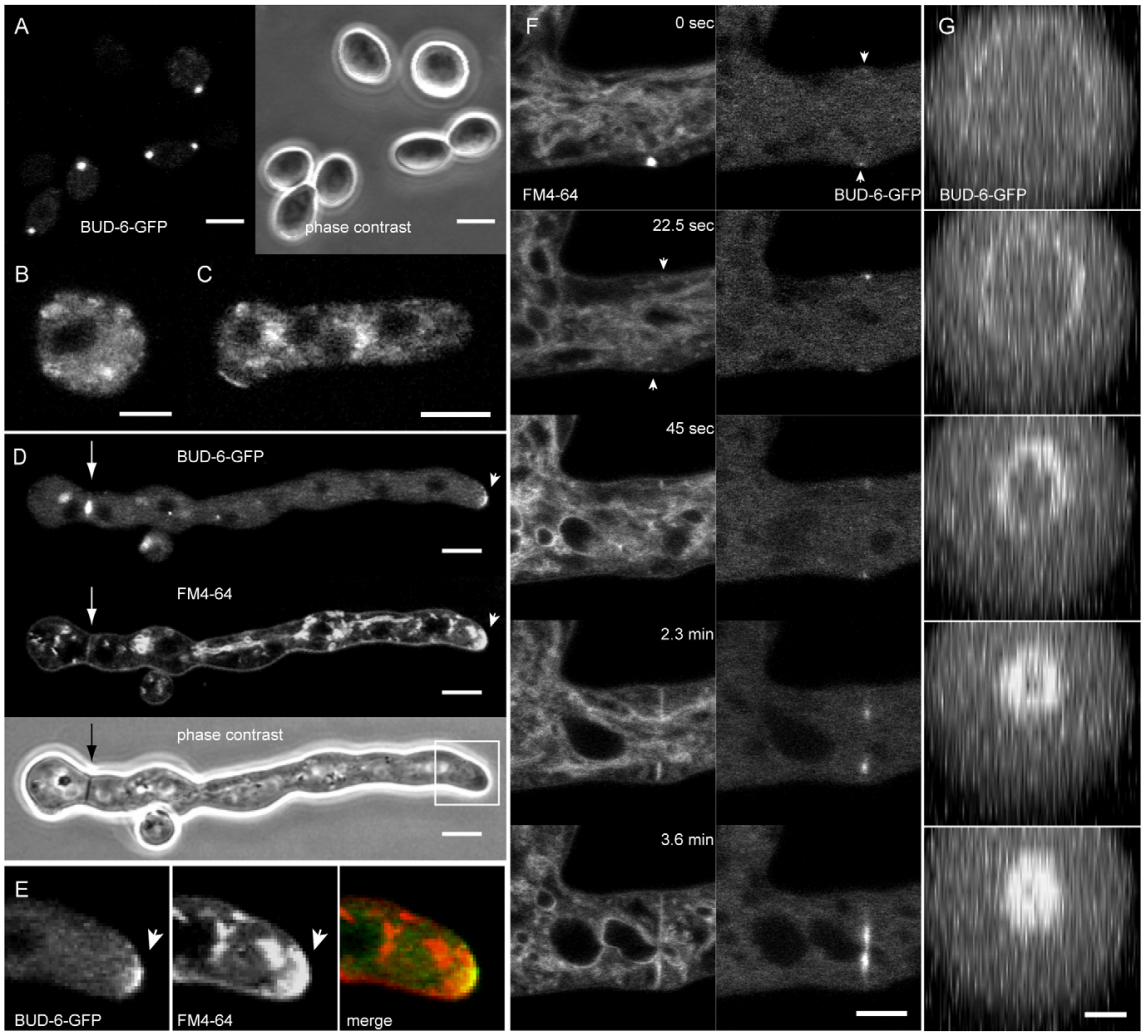
BUD-6 localization in conidia, conidial germlings and sites of septum formation. (**A**) Intense and locally defined clusters of BUD-6 were localized at the cell poles of dormant macroconidia. Scale bars, 5 µm. (**B–C**) Specific recruitment of BUD-6 during isotropic expansion, cell symmetry breaking and germ tube outgrowth was not observed. Scale bars, 2.5 µm. (**D**) Localized recruitment of BUD-6-GFP to apical caps of growing germ tubes (arrowhead), as well as to septa (arrow), occurred in germlings ≥35 µm in length. FM4–64 stained vesicle clusters were observed at the same positions. (**E**) Enlarged view of the germ tube tip highlighted in (D). The merged image shows that BUD-6-GFP fluorescence only partially colocalizes with the FM4–64 stained apical cap which extends over a larger crecent. (**F**) Staining with FM4–64 revealed that BUD-6 recruitment to the incipient septation site preceded plasma membrane invagination (arrowheads), and that it constantly remained associated with the leading edge of the closing septum. Scale bar, 5 µm. See Movie S1 for time course sequences. Note: the bright FM-4–64 stained spot appearing at time point 0 sec does not colocalize with the cortical BUD-6 accumulation indicated by the arrowhead. (**G**) Reconstruction of BUD-6-GFP localization at the inner perimeter of the closing septal pore. Scale bar, 2.5 µm.

In dormant conidia weak BUD-6 fluorescence could be observed in the cytoplasm and as strongly fluorescent, discrete clusters at one or both cell poles (Figure 1A). Upon hydration and isotropic cell expansion, BUD-6 fluorescence became dispersed throughout the cytoplasm (Figure 1B), but discrete cortical accumulations at incipient site of germ tube emergence could not be observed. Apart from occasional concentration in what appeared to be membranous compartments, and obvious exclusion from nuclei, the overall fluorescence pattern in the cytoplasm did not significantly change upon cell symmetry breaking and germ tube protrusion (Figure 1C).

A more focused and specific recruitment to the growing germ tube tip occurred when the germling reached a length of about 35 µm (Figure 1D). In that case, BUD-6-GFP formed an apical cap with the highest concentration of fluorescence at the very tip, which colocalized with an accumulation of vesicles stained with FM4–64 (arrowhead in Figure 1D; enlarged view shown in Figure 1E). Notably, the apical area stained by FM4–64 localizes over a wider crescent than that labelled with BUD-6-GFP. Taken together, these findings are consistent with a role for BUD-6 in the maintenance of germ tube polarity rather than its establishment.

### BUD-6 was part of the contractile acto-myosin ring during septum formation

BUD-6 participated in septum formation in germlings (arrow in Figure 1D) and mature hyphae (Figures 1F and G). Small clusters of BUD-6 fluorescence localized to the incipient site of septum formation shortly before FM4–64 staining indicated plasma membrane invagination (arrowheads Figure 1F, Movie S1). Subsequently, BUD-6-GFP remained associated with the leading edge of the progressively inward growing septum, and finally concentrated as a ring surrounding the septal pore (Figure 1G). This data suggests, that BUD-6 might be part of the landmarking machinery that determines the site of septation, and clearly is part of the contractile actomyosin ring (CAR) driving septum constriction [22], [26], [27]. Interestingly, upon the completion of a septum a ring of BUD-6 persisted for more than four hours, suggesting additional roles of the protein at the inner perimeter of the septal pore.

### BUD-6 and SPA-2 participated in CAT-mediated cell fusion

**Figure 2:**
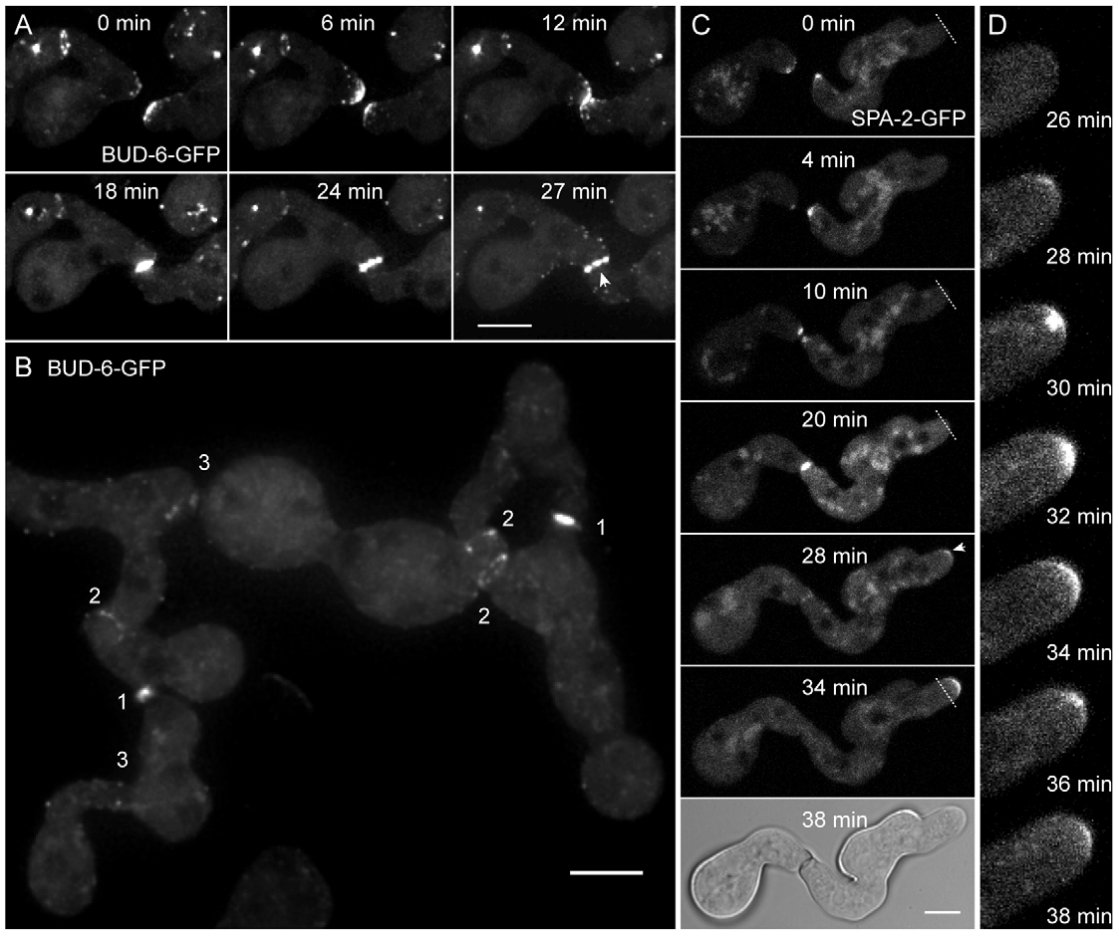
BUD-6 and SPA-2 recruitment during CAT-mediated cell fusion. (**A**) Clusters of BUD-6-GFP became recruited to CAT tips, concentrated at the incipient fusion site upon contact, and formed an opening ring of fluorescence during fusion pore formation (arrowhead). See Movie S2 for time course sequences. Scale bar, 5 µm. (**B**) Typical changes in the BUD-6 localization pattern accompanied distinct stages of the cell-cell fusion process in germling networks: (1) pronounced accumulation shortly after CAT attachment, (2) ring formation during fusion pore opening, and (3) BUD-6 disappearance shortly after cytoplasmic continuity was established. Scale bar, 5 µm. (**C**) Z-projections of confocal optical sections through conidial germlings expressing SPA-2-GFP during CAT homing and fusion. Shortly after cytoplasmic continuity was successfully established and SPA-2 disappeared from the fusion site, a new cluster of SPA-2 became recruited to the germ tube tip (arrowhead) that resumed polarized growth (transient arrest of germ tube growth during cell fusion, and resumed tip extension post-fusion is indicated with dotted line). Scale bars, 5 µm. See Movie S3 for full time sequence. (**D**) Shows an enlarged view of BUD-6 recruitment during the continuation of germ tube tip growth as shown in (C).

A marked increase in localized recruitment of BUD-6 occurred during CAT-mediated cell fusion between conidial germlings (Figure 2A, Movie S2). During CAT homing cortical clusters of BUD-6 formed at the tip apex and coalesced at the incipient fusion point as soon as both CATs made contact. BUD-6 fluorescence concentrated at the site of fusion pore formation, was present as a ring during pore opening and disappeared shortly after cytoplasmic continuity had been established. Changes in the BUD-6 fluorescence pattern in germling networks marked the different stages of the cell-cell fusion process (Figure 2B). The polarisome scaffolding protein SPA-2 showed very similar dynamics during CAT-mediated cell fusion (Figure 2C), including the dispersal of the protein. In the example shown, GT extension transiently arrested during cell fusion, but as soon as cytoplasmic continuity has been established, a new cluster of SPA-2 assembled at the germ tube tip (arrowhead in Figure 2C), and polarized tip extension resumed (Figure 2D and Movie S3). Taken together, BUD-6 and SPA-2 are both part of the polarity machinery organized in an apical cap that drives CAT homing, and both are localized at the site of CAT fusion.

### BUD-6 showed a unique localization pattern forming a subapical cloud around the Spitzenkörper

**Figure 3:**
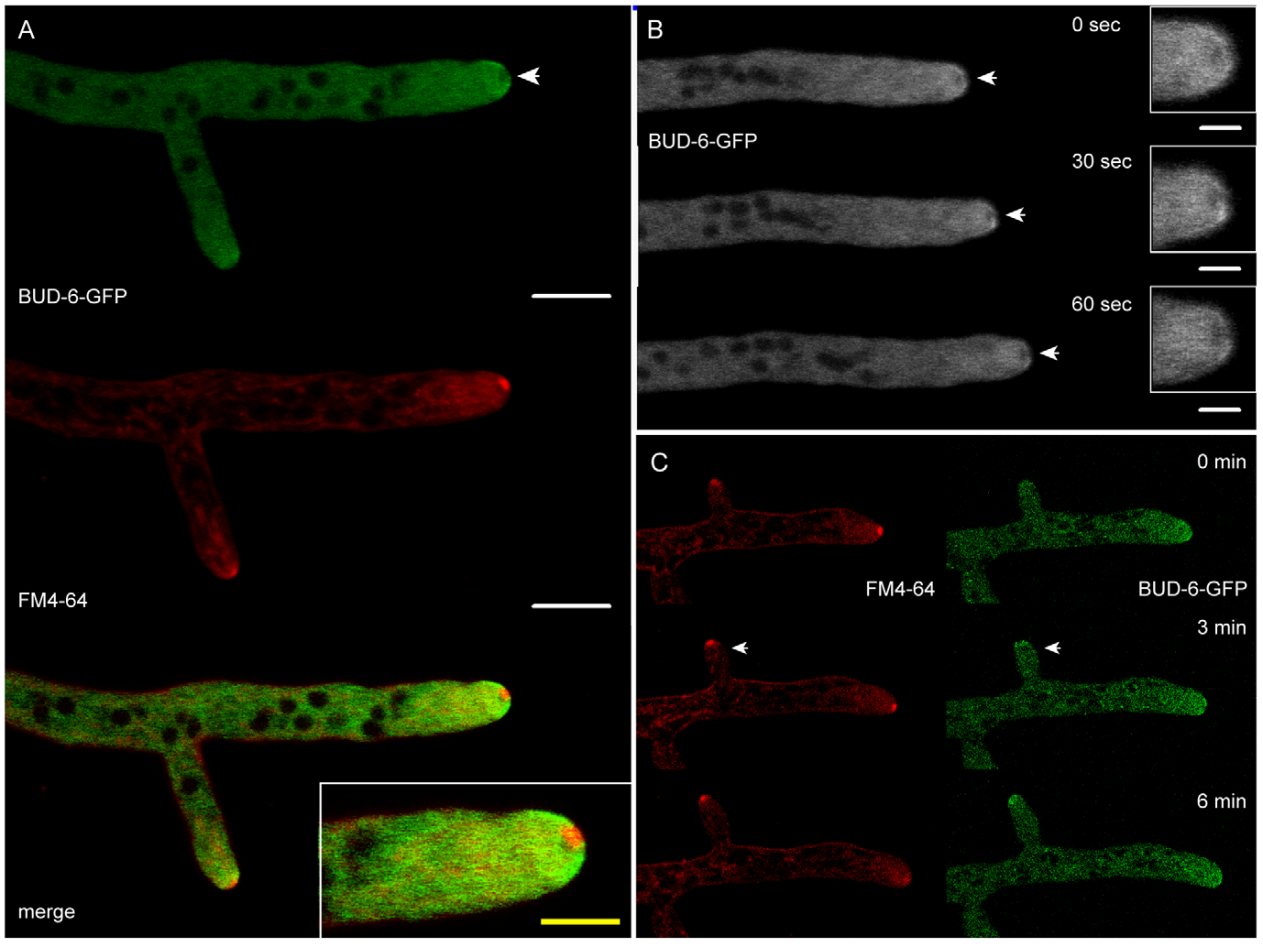
BUD-6 recruitment to polarized growing mature hyphal tips and branches. (**A**) BUD-6-GFP accumulated as a subapical cloud with a distinct exclusion zone at the very tip apex (arrowhead). FM4–64 staining revealed that this exclusion zone was occupied by the Spk. Scale bars, 10 µm; in inset, 5 µm. (**B**) Recording BUD-6-GFP dynamics during hyphal tip growth showed that the BUD-6-GFP exclusion zone (arrowheads and inset for magnified view) consistently remained at the extending tip apex. Scale bars, 10 µm. See Movie S4 for time course sequences. (**C**) Recruitment of BUD-6 to incipient lateral branch points was not observed. Detectable BUD-6 accumulation occurred after the Spk became stained with FM4–64 in newly established branches (arrowheads).

In mature hyphae a very distinct localization pattern was observed for BUD-6. Instead of localizing to an apical cap or to the Spk, as seen for many other components of the polarity machinery, BUD-6 fluorescence exclusively appeared in a heterogeneous, subapical cloud around the Spk (Figure 3). BUD-6 was more concentrated in some regions of the BUD-6 cloud and the localization of these brighter, more fluorescent regions changed with time indicating fast dynamics of the protein (Figure 3B). The region at the hyphal apex which is usually occupied by the Spk was completely free of BUD-6 fluorescence (Figure 3A), but clearly visible with FM4–64 staining. Figure 3B shows the dynamic behaviour of BUD-6 in the apical dome during polarized tip growth (see Movie S4). Cortical accumulation of BUD-6 at incipient branch points was never observed, and detection of apical BUD-6-GFP signal in branch tips coincided with but did not precede the appearance of a FM4–64-stained vesicle cluster (Figure 3C). This observation is in line with the finding from conidial germlings that BUD-6's major role is in the maintenance of polarity but not its establishment.

### Re-establishment of tip growth after physical injury involved recruitment of BUD-6 and SPA-2 to the septal plug and regenerating hyphal tip

**Figure 4:**
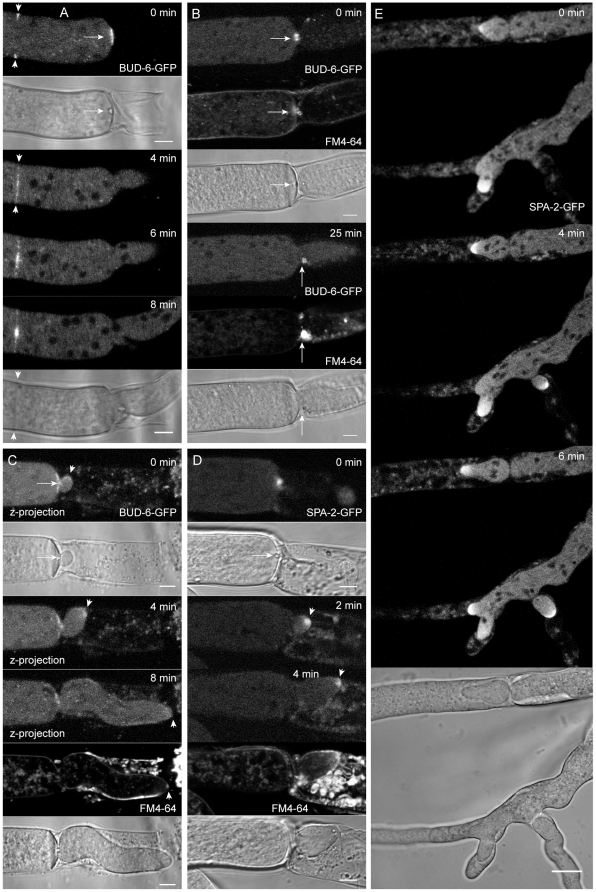
BUD-6 and SPA-2 concentrated at the septal plug prior to reinitiation of polarized tip growth. (**A**) Upon physical injury a Woronin body occluded the septal pore which was surrounded by pre-existing BUD-6 fluorescence (arrow). At the same time, a new septum was being initiated 25 µm further back (arrowheads), again leaving BUD-6 at the septal pore after its completion. (**B**) At the onset of repolarization from the septum at the severed end of the hypha, BUD-6-GFP fluorescence became concentrated at the sealed pore, in the vicinity of the Woronin body and within an accumulation of lipophilic, possibly membranous, material stained by FM4–64 (arrows). See Movies S5 for full sequence. (**C**) The BUD-6 cluster often remained in place while the new tip emerged (arrowhead). Subsequently and coinciding with the condensation of a recognizable Spk, apical BUD-6 fluorescence became increasingly diffuse (arrowheads). See Movie S6 for full sequence. (**D**) SPA-2 rapidly became recruited to the occluded septal pore forming an intense spot associated with the Woronin body (arrow). The protein did not reside at consolidated septal pores, but rather relocated into an apical cap of the emerging tip (arrowhead) which accompanied extension of the new hyphae (**E**). See Movie S7 for full sequences of D. Scale bars A to D, 5 µm. Scale bar E, 10 µm.

To test potential functions of the persisting ring of BUD-6 at completed septal pores (Figure 1H) we performed wounding assays that trigger immediate pore occlusion with Woronin bodies, subsequent consolidation of the sealed pore, and the rapid reinitiation of tip polarity from the severed end (for a recent review see Jedd, 2010). Within two minutes of a hypha being physically injured, a Woronin body occluded the septal pore, and BUD-6-GFP that was originally localized around the septal pore (Figures 1F–H) disappeared within 4 min once a new hyphal tip emerged from this septum (Figure 4A). Often further recruitment of BUD-6 into a cortical ring preceding and accompanying the formation of a new septum 20–30 µm behind the sealed septum could be observed (Figure 4A). Interestingly, coinciding with the onset of reinitiation of polarized tip growth from the septum closest to the severed end of the hypha, BUD-6 became focussed at the septal plug. This occurred in parallel with the recruitment of FM4–64-stained lipophilic, possibly membranous, material in the vicinity of the Woronin body (Figure 4B). This plug complex, consisting of Woronin body, BUD-6 and FM-64 stained material, either became pushed aside by the emerging tip (Figure 4B, 25 min), or remained in place (Figure 4C). In any case, while one population of BUD-6 remained at the plug complex, another population became recruited to an apical cap of the regenerating hyphal tip (Figure 4C). Subsequently and coinciding with the appearance of a FM4–64 stained Spk, apical BUD-6 fluorescence became increasingly diffuse (arrowheads in Figure 4C, 8 min), suggesting rearrangement of the protein into a subapical cloud within a time window of about 10 minutes. Interestingly, SPA-2 which generally does not reside at completed septal pores [59] also became recruited to the plug, and colocalized as intense spot in the vicinity of the Woronin body (Figure 4D). SPA-2, however, did not remain associated with this site, but rather relocated to an apical cap at the emerging and extending hyphal tip (Figure 4D, 2 and 4 min; Movies S5, S6, S7). Together, these observations indicate a role for BUD-6 and SPA-2 in the completion of the septal plug, and the rapid reestablishment of polarized tip growth.

### Recruitment of BUD-6 and SPA-2 during vegetative hyphal fusion in the mature colony followed identical dynamics as observed during CAT-mediated cell fusion

**Figure 5:**
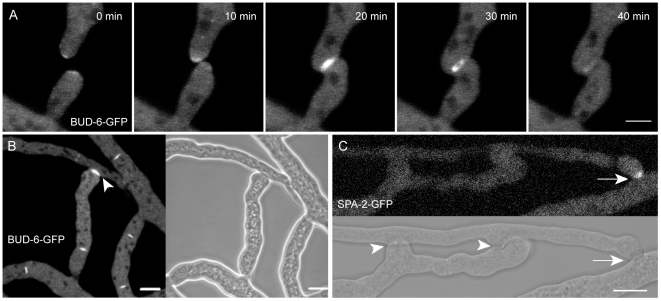
BUD-6 dynamics during vegetative hyphal fusion. (**A**) BUD-6 became recruited to the tips of homing fusion hyphae, then concentrated at the attachment point and surrounded the opening fusion pore. Shortly after the pore was fully established BUD-6 fluorescence disappeared from this site. Scale bar, 5 µm. See Movie S8 for time course sequence. (**B**) Transient BUD-6 fluorescence accumulated at incipient fusion sites in the mature colony (arrowhead) and persistent BUD-6 signal at septal pores (all other fluorescently marked sites). Scale bar, 10 µm. (**C**) SPA-2-GFP became recruited to vegetative hyphal fusion sites (arrow). As it was never seen at completed fusion connections (arrowheads) it must follow the transient dynamics of BUD-6 in this context. Scale bar, 10 µm.

Apical clusters of BUD-6-GFP became recruited to homing tips of fusion hyphae in the mature colony, suggesting the participation of BUD-6 function in vegetative hyphal fusion (VHF). As seen during germling fusion (Figure 2), BUD-6 fluorescence peaked at the incipient fusion site upon cell wall attachment (Figure 5, Movie S8). A ring of fluorescence that increased in diameter could be observed during fusion pore formation, and then gradually disappeared after cytoplasmic continuity was established (Figure 5, 32 min onwards). Thus, BUD-6 did not persist at the completed fusion site as observed upon septum formation (Figure 5B). SPA-2-GFP was also found to accumulate at VHF sites (Figure 5C), and exhibited the same dynamics as BUD-6. Taken together, the BUD-6 VHF localization pattern is distinct from its dynamics during septum formation, and together with the fact that SPA-2 participates in VHF – but not septum formation – provides further evidence that the two processes are distinct from each other.

### BUD-6 participated in cytokinesis during conidiogenesis, and persisted for several days at secondary septa after cell separation

**Figure 6:**
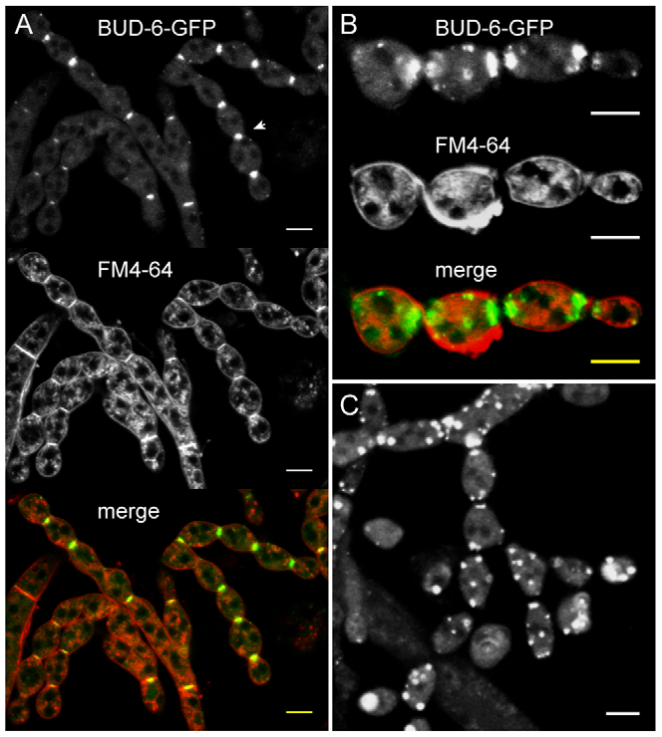
BUD-6 accumulation during conidiogenesis. (**A**) BUD-6-GFP accumulated at septation sites in developing macroconidiophores (arrowhead). Scale bars, 10 µm. (**B**) In cytologically separated, but physically still attached conidia, BUD-6 fluorescence persisted at the cell poles; either at both or only at one pole in case of the terminal conidium. Scale bars, 10 µm. (**C**) In addition to strong fluorescence at the cell poles, bright clusters of BUD-6-GFP also accumulated at other locations of the cell cortex. Scale bar, 5 µm.

In line with the findings of BUD-6 behaviour during septum formation (Figures 1F and G, and 5B), persistent BUD-6-GFP fluorescence was also localized to sites of cytokinesis in developing conidiophores (Figure 6). In addition, in cytologically separated but physically still attached conidia, BUD-6 fluorescence persisted at the cell poles; either at both or only at one pole in case of the terminal conidium (Figure 6B). Strongly fluorescent clusters of BUD-6-GFP of unknown function were also distributed at the cell cortex of attached macroconidia at this stage (Figure 6C). With progressing age of the developing colony the percentage of conidia retaining cortical BUD-6 clusters decreased (Figure S3).

### Characterization of Δ*bud-6*

Homokaryotic Δ*bud-6* strains were successfully generated through backcrossing of the heterokaryotic Δ*bud-6 mat a* gene deletion strain obtained from the FGSC with a Δ*sad-2 mat A* strain incapable of meiotic silencing (Table 2), and with the selection of ascospore progeny on hygromycin B medium. For 15 out of the 20 selected clones (75%), the phenotypic change cosegregated with hygromycin B resistance, indicating that the resulting mutant phenotype was caused by the targeted gene deletion and not due to unintended rearrangements introduced during sexual reproduction elsewhere in the genome. Absence of the native *bud-6* locus from the genome was confirmed by PCR (Figure S1). The homokaryon selection procedure using crossing has been repeated once, and confirmed by vegetative selection of single conidia. Both selection strategies resulted in hygromycin B resistant colonies with identical mutant phenotypes.

**Table 2:** *N. crassa* strains used in this study.

Strain	Genotype	Source
Wild type N150	wt; mat A	FGSC # 9013
Δ*mus51*	Δ*mus-51::bar^+^; his^−3^*; mat A	FGSC # 9717
Δ*bud-6* (heterokaryon)	Δ*bud-6*::*hph^+^*, Δ*mus-51*:: *bar*+*bud-6*+*mus51;* mat a	FGSC # 11424
Δ*sad-2*	Δ*sad-2*::*hph^+^,* mat A	Shiu *et al.*, 2006
Δ*bni-1* (heterokaryon)	Δ*bni-1*::*hph*, Δ*mus-51*:: *bar*+*bni-1*+*mus51;* mat a	FGSC # 11490
Δ*bni-1*	Δ*bni-1::hph^+^*, Δ*mus-51*:: *bar^+^, mat a*	This study
Δ*spa-2*	Δ*spa-2*::*hph^+^,* mat A	FGSC # 11140
Δ*spa-2*	Δ*spa-2*::*hph^+^*, mat a	FGSC # 11141
NCLAP5-1	*his^−3^*+*Pccg-1::spa-2-sgfp;* mat A	Araujo-Palomares *et al.*, 2009
NECL46-9	Δ*mus-51::bar^+^; his-3* ^+^::*Pccg-1*::*bud-6-sgfp;* mat A	This study
NECL47-5	Δ*mus-51::bar^+^; his-3* ^+^::*Pccg-1::bud-6-mchfp;* mat A	This study
NECL48-8	Δ*bud-6::hph^+^,* mat a	This study
*bni-1-sgfp*	Δ*bni-1::hph^+^, his-3* ^+^::*Pccg-1::bni-1-sgfp;* mat a	Justa-Schuch *et al.*, 2010

### BUD-6 was required for normal colony development, maintenance of hyphal morphology, septum formation and conidiogenesis

**Figure 7:**
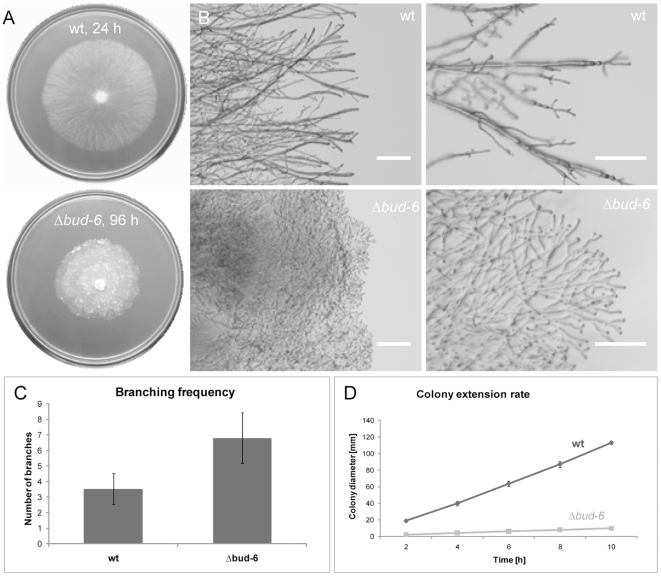
Loss of BUD-6 resulted in a reduced colony extension rate and hyperbranching. (**A**) Hyperbranching and polar extension defects resulted in very slowly and extremely dense developing mycelial colonies of Δ*bud-6*, in comparison to the wild type after 24 hours of incubation. (**B**) Hyphal morphology of wild type and Δ*bud-6* at the colony margin. Scale bars, 0.5 mm and 0.25 mm, respectively. (**C**) Quantification of branching frequency, which on average was more than doubled in the mutant compared to the wild type. (**D**) Comparison of average colony extension speed between Δ*bud-6* and wild type.

**Figure 8:**
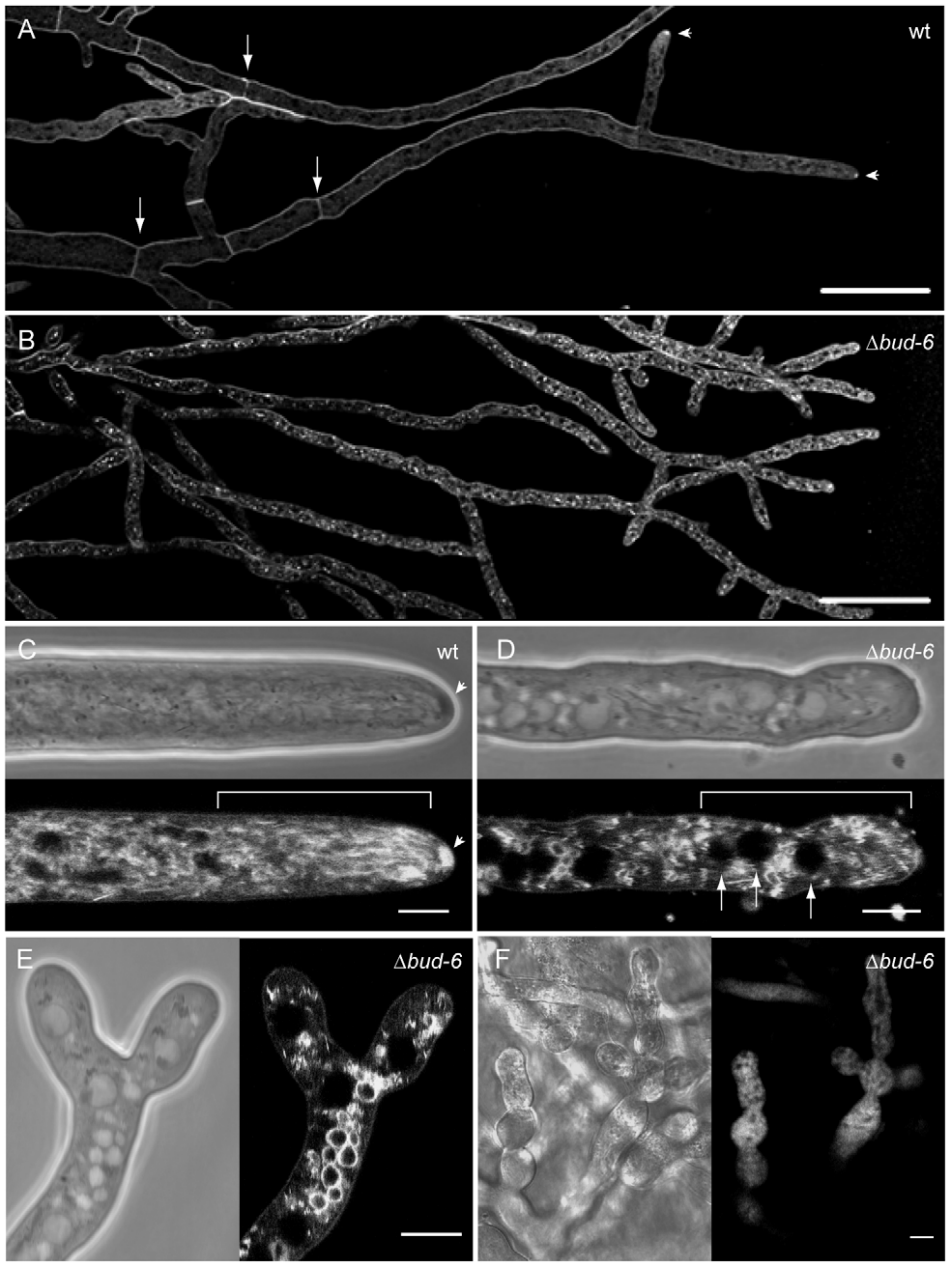
BUD-6 was required to organize the polarized growth apparatus at the hyphal tip. (**A and B**) Comparison of FM4–64 staining pattern of mature hyphae at the leading edge of the colonies in the wild type and Δ*bud-6* mutant confirmed the absence of septa in the mutant (arrows in A indicate septa in the wt), as well as the absence of the Spk at the hyphal tips of Δ*bud-6* (arrowheads in A point toward wt Spk). Scale bars, 5 µm. (**C and D**) Close up of the apical and subapical area of polarized growing mature hyphae of wt and Δ*bud-6*. The arrowheads in C indicate the Spk, which shows up as a dark sphere in the phase contrast image and was brightly stained by FM4–64. No such structure was observed in hyphae of the Δ*bud-6* mutant. The squared bracket marks the subapical nuclear exclusion zone in the wild type, which is not established in the Δ*bud-6* mutant. Here, nuclei (arrows) reach further up into the hyphal tip (also seen in E). Scale bars, 5 µm. (**E**) Apical branching and lack of hyphal tip organization in Δ*bud-6*. Scale bar, 5 µm. (**F**) Immature and malformed conidiophores in Δ*bud-6*. Scale bar, 10 µm.

Due to multiple morphological defects, loss of BUD-6 resulted in mutant strains unable to establish normal colony architecture (Figure 7A and B). Defects included, hyperbranching, lack of hyphal differentiation - including primary and secondary branches and fusion hyphae - lack of septa, and incomplete conidiogenesis. In comparison to the wild type, branching frequency was more than doubled in Δ*bud-6* mutants (Figure 7C), and radial colony extension was decreased by a factor of 10 or more (Figure 7D), resulting in very dense, slow growing and aconidiate colonies. FM4–64 staining of Δ*bud-6* hyphae confirmed the absence of septa and revealed another highly interesting phenotype; the absence of the Spk (Figures 8B–E). Despite the apparent loss of organized assembly of the polarized tip growth apparatus, evident e.g. by the lack of a nuclear exclusion zone (Figure 8D), which normally appears distal to the Spk in the wild type (Figure 8C) [60], or frequent apical branching (Figure 8E), polarized extension of hyphae, however, was still possible. In contrast to most other membranes, such as the plasma membrane, endocytic vesicles or vacuolar membranes, application of the lipophilic dyes FM1–43 or FM4–64 does not stain the nuclear envelope in *N. crassa* [54], [61]. Therefore, nuclei appear negatively stained as black subspherical objects without a fluorescent border in the otherwise highly fluorescent membranous background of the cytoplasm. In some instances, conidiophore-like aerial hyphae could be observed in older parts of the mycelium which attempted to differentiate conidia through constriction, but due to the apparent block in cytokinesis were never completed (Figure 8F). Taken together, the phenotypic defects mirrored the multifunctional role of BUD-6 implicated by its subcellular localization: in apical caps in growing germ tubes, CATs and regenerating tips; as subapical cloud in mature hyphal tips and branches; at forming septa and persistent at septal pores, as cytokinetic rings during conidiogenesis; and at sites of VHF.

### Characterization of Δ*bni-1*

A key effector of polarisome activity in yeast is the formin Bni1, which stimulates F-actin nucleation in a Bud6-dependent manner [32], [41]. To investigate the functional relationship of the only formin homolog identified in *Neurospora*, BNI-1, to the filamentous fungal polarisome, we sought to characterize the corresponding gene deletion mutant (Table 2), and analyze the subcellular dynamics of BNI-1-GFP [62] in greater detail, and in relation to SPA-2 and BUD-6.

### Deletion of the formin gene *bni-1* phenocopied Δ*bud-6* to a large extent

**Figure 9:**
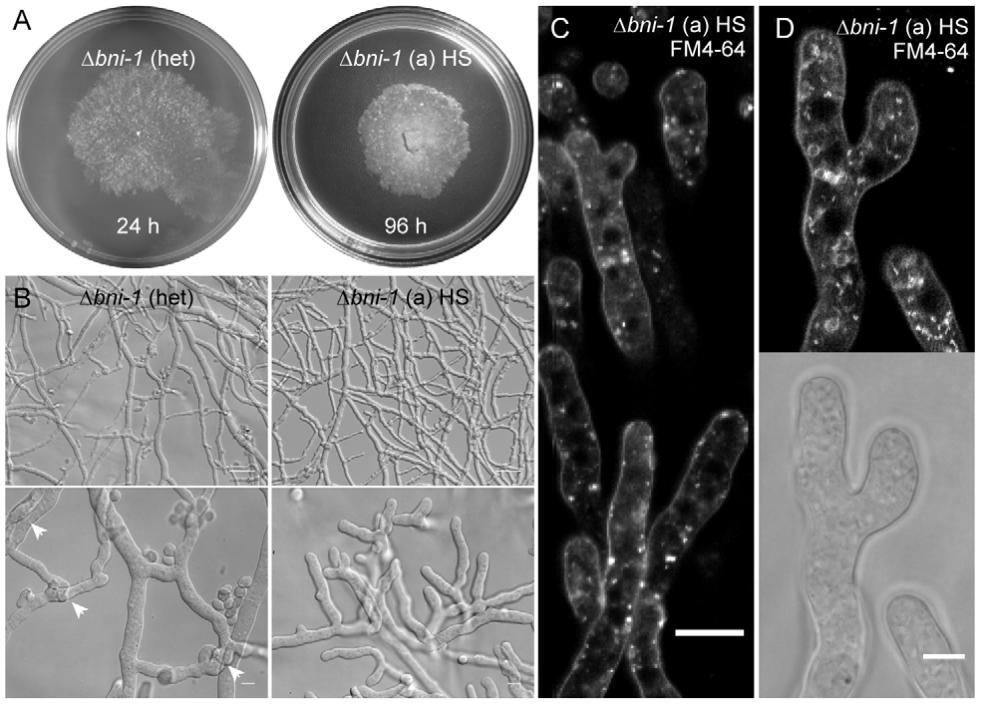
A Δ*bni-1* strain generated through vegetative homokaryon selection phenocopied growth defects of Δ*bud-6*. (**A and B**) Wild-type like phenotype of the heterokaryotic Δ*bni-1* strain FGSC11490, including conidia and septa (arrows). Scale bars, 50 µm and 10 µm respectively. (**C**) The lack of septa in the homokaryotic Δ*bni-1* strain was confirmed by FM4–64 staining. Scale bar 50 µm. (**D**) FM4–64 staining also confirmed the absence of an organized apical tip growth apparatus, including the Spk. Scale bar, 10 µm. These defects closely resembled phenotypic key features of Δ*bud-6* (Figures 7 and 8).

Genetic deletion of *bni-1* has been reported to be lethal because viable ascospores could not be recovered from sexual backcrosses between the Δ*bni-1* heterokaryon (FGSC11490) and the wild type [62]. In an alternative approach, we attempted to remove wild type nuclei carrying the native copy of *bni-1* from the heterokaryotic gene deletion strain vegetatively, i.e. through repeated isolation of monosporic microcolonies on selection medium. This method quickly, i.e. within one generation, resulted in strains with strong phenotypic defects very similar to those observed for Δ*bud-6* (Figure 9). Phenotypic alterations of the homokaryon-selected strains included, apical branching, the lack of hyphal differentiation, and the absence of septa, conidiophores and consequently conidia. PCR genotyping confirmed the correct exchange of the native gene locus against the knock-out cassette, as well as absence of *bni-1* from the whole genome (Figure S1B). The strain purification and verification process was reproducible, suggesting that the observed phenotype was indeed the result of the exclusive deletion of the *bni-1* locus, and not due to random mutations or other unintended alterations within the genomes of these strains.

**Figure 10:**
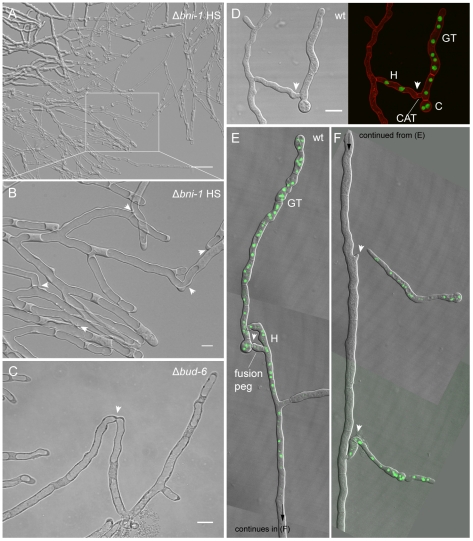
Δ*bni-1* and Δ*bud-6* strains displayed derepressed hyphal fusion at the colony edge. (**A**) The lack of septa in Δ*bni-1* resulted in extensive accumulation of vacuoles at the leading edge of the colony. Scale bar, 50 µm. (**B**) Hyphal fusion at the leading edge of the colony, which is usually suppressed in the wild type through apical dominance, was observed in Δ*bni-1*. Several fusion pores are indicated with arrowheads. Scale bar, 10 µm. See Movie S9 for time course sequence showing vacuolar passage through fusion connections. (**C**) After closer inspection, the same phenotype could be observed at the leading edge of Δ*bud-6* colonies. Scale bar 10 µm. (**D–F**) In the wild type, derepression of hyphal fusion at the leading edge occurred in the presence of conidial germlings. The establishment of cytoplasmic continuity – here visualized through the transfer of nuclei fluorescently labeled with histone H1-GFP (green) from conidial germlings into mature hyphae - involved either CAT-mediated cell fusion (D), or fusion pegs from the mature hypha (E and F) induced through the presence of conidial germlings. Arrowheads indicate fusion sites; C denotes the spore body; GT denotes the germ tube; H denotes mature hypha. Note that fluorescently labeled nuclei originating from the germling have only migrated into the upper part of the unlabeled wild type hypha. The arrowheads in (F) mark fusion pegs emerging from the mature hypha. Scale bar in D, 10 µm.

In line with findings in the Δ*bud-6* strain, FM4–64 staining of hyphae at the leading edge of Δ*bni-1* colonies confirmed the lack of septa, and most notably also included the lack of any functional organization of the tip apex; evident by the absence of the Spk and nuclear exclusion zone (Figure 9C and D). Interestingly, the absence of septa resulted in the pronounced accumulation of vacuoles at the leading edge of a colony (Figure 10A and B). Despite the absence of differentiated fusion hyphae in the mature mycelium, Δ*bni-1* was able to undergo hyphal fusion. Unexpectedly, hyphal fusion occurred at the very colony edge (Figure 10B, Movie S9); a feature not seen in the wild type, in which fusion is restricted to the subperiphery and beyond, but generally suppressed at the colony margin [63]. Upon closer inspection, we also found evidence for this phenotype at the colony periphery of Δ*bud-6* (Figure 10C).

The only example of derepression of hyphal fusion at the colony periphery in the wild type, occurred as a result of interactions with conidial germlings (Figure 10D to F). The establishment of cytoplasmic continuity between conidial germlings and mature hyphae, involved either CATs protruded from germlings (Figure 10C), or the induction of fusion pegs from mature hyphae through the presence of germlings (Figure 10D and E).

Taken together, the morphogenetic defects of Δ*bni-1* phenocopied those of Δ*bud-6* to a large extend, indicating that both proteins together operate in similar cellular contexts. To investigate these further we compared the subcellular dynamics of BNI-1-GFP with those of the other two polarisome components (BUD-6 and SPA-2) during the same set of morphogenetic processes.

### BNI-1 localized to sites of cell symmetry breaking, polarized germ tube growth, CAT-mediated cell fusion, vegetative hyphal fusion and septum formation

**Figure 11:**
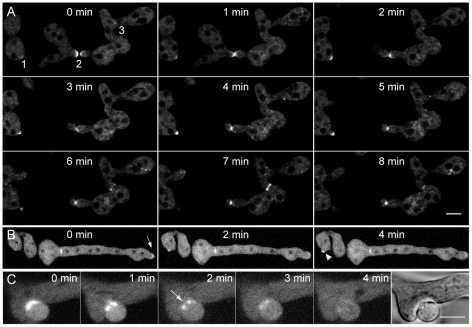
BNI-1 recruitment to sites of polarized growth, septum formation and cell fusion. (**A**) In conidial germlings recruitment of fluorescently labeled BNI-1 was observed during three different cellular processes. (1) During cell symmetry breaking BNI-1 appeared at the cell cortex. (2) During CAT-mediated cell fusion, very bright accumulations of BNI-1 could be seen at the tips of interacting CATs. Due to a spore torque response upon cell-cell attachment, the left cell moved out of the focal plane. This movement is commonly observed when imaging germling fusion in liquid medium. (3) During septum formation BNI-1 was part of contractile actomyosin rings. Scale bar, 5 µm. (**B**) BNI-1 also accumulated in apical crescents at growing germ tube tips (arrow). Recruitment to septal pores and a new sites of cell symmetry breaking (arrowhead) showed up as well. Also note that BNI-1 is associated with septum formation (asterisk) at the base of the germ tube. (**C**) Consistent with its participation in CAT-mediated cell fusion, BNI-1 also became recruited to sites of vegetative hyphal fusion, and disappeared shortly after cytoplasmic continuity was established. The arrow marks the opening fusion pore. Scale bar, 5 µm.

During CAT-mediated cell fusion BNI-1-GFP dynamics followed those of BUD-6 and SPA-2 (Figure 2). The formin became recruited to homing CAT tips, focused at the attachment site and disappeared after cytoplasmic continuity was successfully established (Figure 11A, Movie S10). In contrast to BUD-6 and SPA-2, BNI-1 also became recruited to the cell cortex prior to cell symmetry breaking and remained associated with the tip of the forming protrusion (Figure 11A and B). VHF connections within the mycelia network of the Δ*bni-1*::*bni-1-gfp* strain (Table 2) were indistinguishable from the wild type and thus BNI-1's function in VHF was consistent with its role during CAT-mediated cell fusion (Figure 11A). The time sequence in Figure 11C shows that BNI-1-GFP labelled the inner perimeter of the opening fusion pore and disappeared immediately after cytoplasmic continuity was established. Again, following the dynamics observed for BUD-6 and SPA-2 (Figure 5), suggesting that during VHF all three proteins colocalize. As reported earlier for mature hypha [62], BNI-1 furthermore accumulated at incipient septation sites in conidial germlings, forming a constricting ring (Figure 11A). In polarized growing germ tubes BNI-1 accumulated in an apical crescent (Figure 11B). The unique insight from this set of experiments is that BNI-1, at least in the unicellular germling growth phase, is the only one of the three proteins that is clearly involved in the *de novo* establishment of new polarized growth sites.

### In mature hyphae BNI-1 localized to both an apical cap and the Spitzenkörper core

**Figure 12:**
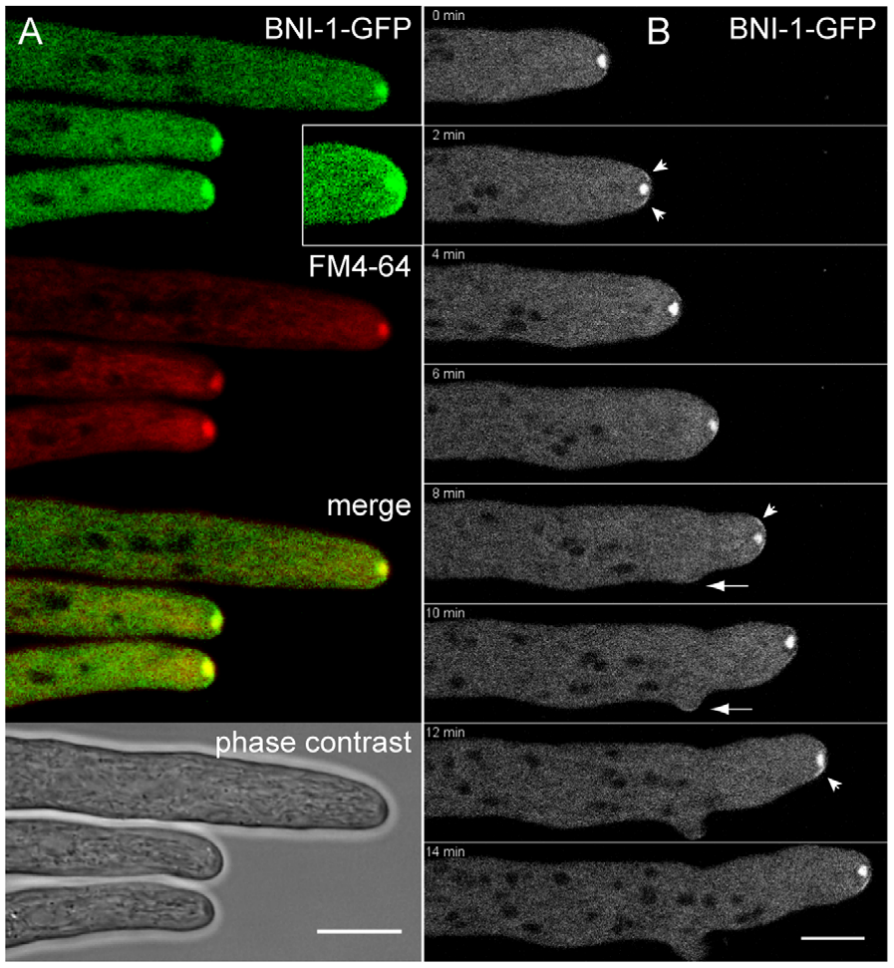
BNI-1 is a constituent of the Spk, but also localized to an apical cap. (**A**) BNI-1-GFP colocalized with the Spk, but also was present in an apical cap (see inset in A). (**B**) Time sequence of BNI-1 dynamics during mature hyphal tip growth and lateral branch initiation. 0–2 min: in the straight growing hyphal tip small crescents (arrowheads) of BNI-1 were located on either side of the Spk. 4–6 min: shortly before branch initiation, tip extension transiently ceased – evident by rounding-off of the tip - and apical BNI-1 fluorescence disappeared. 8–10 min: extension of the main tip resumed with a brighter cluster of BNI-1 at the left hand side of the apex, followed by displacement of the Spk and left-orientation of the tip. A small crescent of formin fluorescence localized to the tip of the emerging branch (arrow). 12–14 min: a brighter cluster of the formin accumulated at the right hand side preceding reorientation of tip extension into this direction. Scale bars, 10 µm.

Further analyses in mature hyphae revealed that BNI-1 not only accumulated in apical clusters as already demonstrated [62], but moreover was a constituent of the Spk (Figure 12A). Relocation of BNI-1 clusters within the apical cap (Figure 12B) preceded Spk displacement and tip reorientation. Formation of a cortical BNI-1 crescent also coincided with lateral branch initiation (Figure 12B), during this event apical BNI-1 clusters associated with the apical plasma membrane transiently disappeared and extension of the main tip paused. This data suggests that targeted activation of the formin at specific locations inside the apical dome might underlie the regulation of directional tip growth, probably by actin-mediated interaction with the Spk. It furthermore confirmed the function of BNI-1 in polarity establishment as already indicated by the findings in conidial germlings (Figure 11).

### BNI-1 became transiently recruited to septal plugs and showed identical dynamics at repolarizing hyphal tips as SPA-2, but only partial spatiotemporal overlap with BUD-6

**Figure 13:**
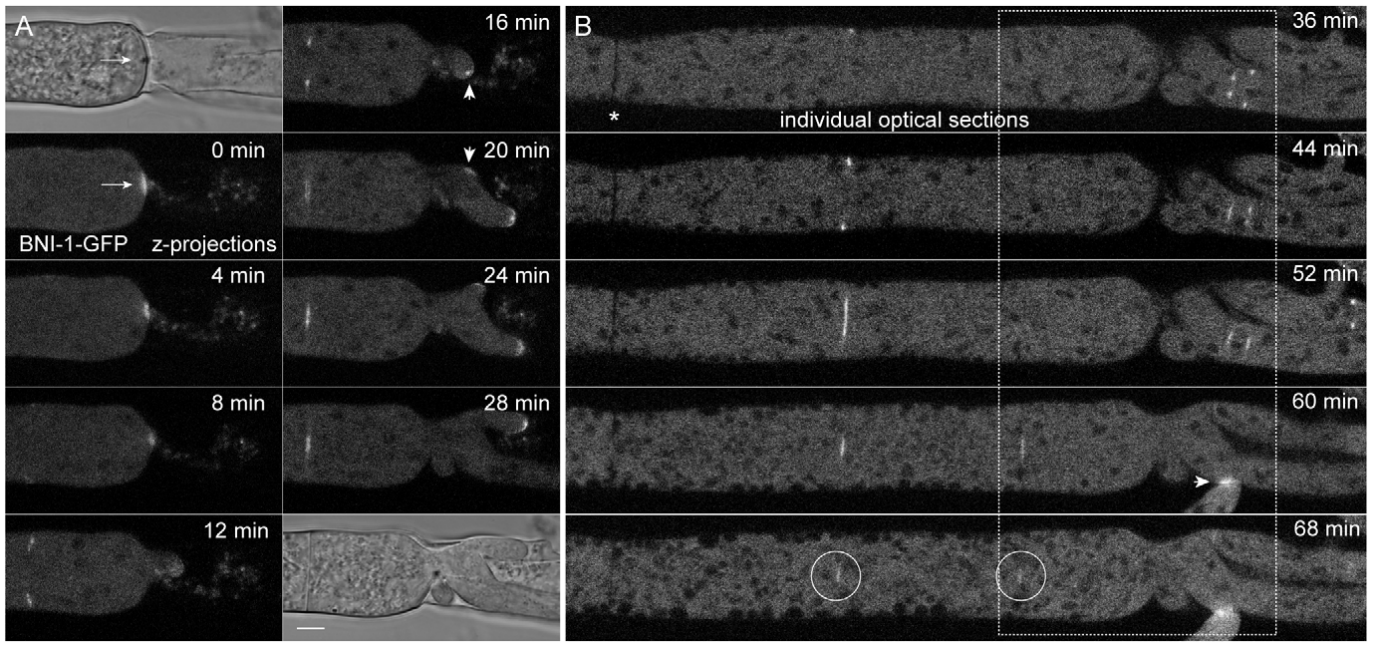
BNI-1 localization during septal plugging, tip repolarization and septum formation. (**A**) Two minutes after damaging leading hyphae BNI-1 became recruited to the septal plug (position of the Woronin body is indicated with an arrow, 0 min). Fluorescence focused into a smaller area from which a new hyphal tip repolarized, and shortly after condensed into an subapical spot (arrowhead, 16 min) with flanking BNI-1 crescents on either side (inset, 20 min). In parallel, a new septum was being formed about 25 µm behind the severed end, and an additional site of polarity was established (arrowhead, 20 min). Scale bar, 5 µm. See Movie S11 for full sequence. (**B**) Continuation of (A) but with an extended field of view including an old septum (asterisk). The part of the hypha shown in (A) is outlined with a dashed box. A selection of individual optical slices shows the formation of several septa. Upon septum completion, BNI-1 gradually disappeared from the septal pore. Barely visible remains are indicated with circles at the 68 min time point. BNI-1 fluorescence was usually not observed at ‘old’ septa (asterisk). Recruitment of BNI-1 to a vegetative hyphal fusion site is indicated by an arrowhead.

About 2 minutes after physical injury had been produced, BNI-1-GFP fluorescence localized around the septal plug closest to the severed end of the hypha (Figure 13A). Shortly after, fluorescence focussed at an even smaller area around the Woronin body from which a new hyphal tip repolarized and extended into the emptied compartment. Different to BUD-6, the formin did not remain associated with the plug complex, but rather migrated with the repolarizing tip apex and later on, in addition, concentrated into a small subapical spot resembling a forming Spk (Figure 13A). It is likely that the formation of this spot coincided with the appearance of a FM4–64-stained vesicle cluster. This however cannot be concluded with certainty as the FM4–64 background fluorescence from cell debris in the damaged compartment hindered its detection (see Movie S11). The dynamics of BNI-1 during this process were identical to those of SPA-2 (Figure 4D), and overlapped with those of BUD-6, which was already present at the septal pore before injury, and thus did not require *de novo* recruitment (Figure 4A and B). Apical recruitment of BNI-1 prior to the outgrowth of a second tip was also observed (Figure 13A), again confirming the presence of BNI-1 during polarity establishment. In parallel, the formation of a new septum was being initiated about 25 µm distal from the severed end (Figure 13A). Notably, at the 20 min time point the segregation of BNI-1 into an apical crescent and subtending spot is indicated in the lower tip (enlarged view in inset). This again fits into the 10 min time window spanning from the reinitiation of tip growth until the architecture of the apical tip growth apparatus adapts for a faster rate of tip extension. Several other septa formed in the vicinity, and in all cases BNI-1 did not reside at septal pores for much longer than an hour. The barely visible residual BNI-1 fluorescence at the two septal pores in the image centre is indicated by circles in Figure 13B. Notably, no BNI-1 fluorescence could be seen at older septa (Figure 13B). This data confirms the transient role of BNI-1 during septum formation as already reported by Justa-Schuch *et al.* (2010), and additionally revealed the rapid recruitment of the formin to sites of polarity re-establishment from the plugged septal pore, as well as the quick (within approx. 10 min) transformation of the tip growth apparatus from an apical cap configuration into an apical crescent with subtending Spk.

### BNI-1 became only transiently recruited to sites of cytokinesis

**Figure 14:**
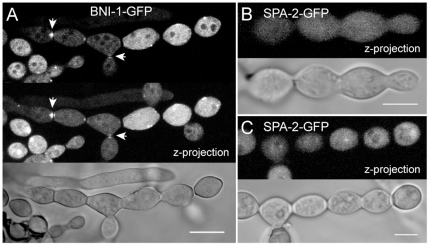
BNI-1 localization during conidiogenesis. (**A**) During cytological compartmentalization of conidiophores BNI-1 localized to forming septa (arrowheads). Apart from occasional cortical clusters no specialized localizations (e.g. to cell poles) of the formin could be observed at mature stages of conidial development. (**B**) In contrast, apart from weak cytoplasmic fluorescence, no specific accumulation of SPA-2 could be observed during conidiophore formation, cytokinesis or (**C**) upon physical separation of mature conidia. Scale bars, 5 µm.

Similar to BUD-6 (Figure 6), BNI-1 fluorescence became recruited to the septation sites in differentiating conidiophores (Figure 14A), indicating that both proteins participate in cytokinesis. However, BNI-1 did not reside at the cell poles/secondary septa as observed for BUD-6 (Figure 6). In line with its transient role during septum formation in mature hyphae, upon physical separation of conidia BNI-1 fluorescence disappeared from secondary septa. Fluorescent accumulations at the cell poles of mature conidia were not observed. Small clusters of BNI-1 randomly distributed at the cell cortex, however, could be observed. In contrast to BNI-1 and BUD-6, but consistent with its absence during septum formation, SPA-2 has so far not been observed to localise to sites of cytokinesis (Figure 14B), nor accumulate elsewhere in the cell upon physical separation and maturation of conidia (Figure 14C). Taken together, this data suggests that BNI-1 only transiently participates in cytokinesis. It thus has no continued function in cell wall fortification in maturing conidia as suggested for BUD-6, and both BNI-1 and BUD-6 function in the absence of SPA-2 during conidiogenesis.

## Discussion

**Figure 15:**
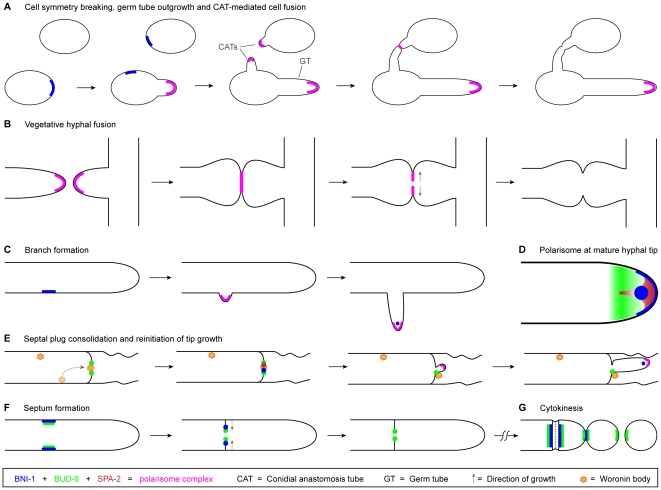
Schematic representation of the subcellular localization and dynamics of the three polarisome components SPA-2, BUD-6 and BNI-1 during key developmental stages of *N. crassa*. (**A**) Prior to the constitution of the entire polarisome complex during germ tube growth and CAT-mediated cell fusion, BNI-1 accumulates at the incipient sites of cell symmetry breaking. (**B**) Equivalent to its dynamics during CAT fusion, the polarisome complex is present during homing and fusion of fusion hyphae, and removed once cytoplasmic continuity is achieved. (**C**) BNI-1 accumulates at the incipient site of branch formation, and together with BUD-6 and SPA-2, subsequently constitutes the complete polarisome complex as an apical crescent at the tip of the emerging branch. Finally, the polarisome adopts the mature hyphal tip configuration (D). (**D**) Configuration of the polarized tip growth apparatus in mature hyphae, including an apical cap and Spitzenkörper core of BNI-1, a fan-shaped distribution of SPA-2 inside the apical dome, and the subapical BUD-6 cloud. (**E**) Septal plugging and consolidation involves the Woronin body and all three polarisome proteins. During repolarization, a polarisome crescent is constituted at the emerging hyphal tip, and ultimately rearranged into its mature form (D). (**F**) Septum formation requires BNI-1 and BUD-6 – but not SPA-2 - as components of the CAR. Upon septum completion, only BUD-6 remains associated with the inner perimeter of the septal pore. (**G**) During cytokinesis BNI-1 and BUD-6 become recruited to the CAR and to forming secondary septa. Upon physical separation of macroconidia BNI-1 disappears, whereas BUD-6 remains at the cell poles. As during septum formation, SPA-2 has no role in this process.

In order to determine during which morphogenetic processes a polarisome complex is constituted, and whether its spatiotemporal dynamics differ from those observed in other fungal species, we generated a comprehensive localization map of the three polarisome components identified in *Neurospora crassa*, We analyzed the subcellular dynamics of the actin-interaction protein BUD-6 and the formin BNI-1 during various morphogenetic transitions that involve changes in the establishment, maintenance and termination of polarized growth, and compared their dynamics to the localization patterns of the *bona fide* polarisome marker SPA-2. Finally, we related the live-cell imaging data to the phenotypic defects observed in the corresponding gene deletion mutants. The key findings of our analyses are summarized in Table 3 and the accompanying schematic diagram in Figure 15.

**Table 3:** Subcellular localization patterns of polarisome components during key developmental stages in *N. crassa*.

	SPA-2	BUD-6	BNI-1	Colocalization‡	Unique roles
**Conidium**	cytoplasmic^1^	cytoplasmic, random cortical clusters, clusters at cell poles	cytoplasmic, random cortical clusters	BUD-6 & BNI-1 in random cortical clusters of unknown function	BUD-6: persistent clusters at cell poles
**CSB**	-	-	recruitment to incipient polarisation sites	-	BNI-1: recruitment during polarity establishment
**Germ tube**	apical cap^2^	apical cap, at septa	apical cap	SPA-2, BUD-6 & BNI-1: apical cap prior to Spk appearance	-
**CAT**	apical cap, transient ring at opening fusion pore	apical cap, transient ring at opening fusion pore, random cortical clusters	apical cap, at opening fusion pore, random cortical clusters	SPA-2, BUD-6 & BNI-1: apical cap during CAT homing and transient ring during fusion pore formation	-
**Hyphal tip**	at Spk and fan-shaped apical dome^2^	subapical cloud, excluded from Spk	in Spk core, apical cap^3^	SPA-2 & BNI-1: partial overlap in the Spk and potentially in the tip apex	BUD-6: subapical cloud, excluded from Spk
**Branching**	apical cap at emerging and growing branch^2^	subapical accumulation coincides with Spk assembly	cortical recruitment to incipient branch site	-	BNI-1: cortical recruitment during branch initiation
**Septal plugging & regeneration of tip growth**	recruitment to septal plug prior to repolarization, apical cap at repolarizing tip	permanently at septal pore, concentrates at septal plug, apical cap at repolarizing tip	recruitment to septal plug prior to repolarization, apical cap at repolarizing tip	SPA-2, BUD-6 & BNI-1: at septal pore and as apical cap at repolarizing tip	BUD-6: remains at septal pore/Woronin body
**Septum formation in mature hyphae**	-2	at incipient septation site, contractile ring, persistence at septal pore	at incipient septation site, contractile ring^3^	BUD-6 & BNI-1: at incipient septation site and part of contractile ring	BUD-6: persistence at septal pore (>4 h)
**VHF**	apical cap, transient ring at opening fusion pore	apical cap, transient ring at opening fusion pore	apical cap, transient ring at opening fusion pore	SPA-2, BUD-6 & BNI-1: transient during all stages of the fusion process	-
**Conidiophore**	-	cytokinetic ring, persistence at secondary septa, random cortical clusters	cytokinetic ring, transient at separation sites, random cortical clusters	BUD-6 & BNI-1: during cell separation	BUD-6: persists at completed cell separation sites/secondary septa
**KO phenotype**	diffuse Spk, irregular hyphal growth^2^	hyperbraching, apical branching, no Spk, aconidiate, aseptate, derepressed VHF at colony edge	hyperbraching, apical branching, no Spk, aconidiate, aseptate, derepressed VHF at colony edge	Δ*bud-6* & Δ*bni-1* phenotypically very similar	-

(-) no specific subcellular recruitment observed/no colocalization indicated/no unique role observed;

‡ Colocalization inferred from identical subcellular localization patterns; CSB = cell symmetry breaking, CAT = conidial anastomosis tube, Spk = Spitzenkörper, VHF = vegetative hyphal fusion (in the mature colony), KO = knock-out (gene deletion) mutant;

References:

1 Araujo-Palmorales, Master Thesis, CICESE, 2007;

2 Araujo-Palmorales et al., 2009;

3 Justa-Schuch et al., 2010.

### Cell symmetry breaking in *N. crassa* is a polarisome-independent process

We found no evidence for BUD-6 or SPA-2 being involved in the establishment of polarized growth in *Neurospora crassa*. Cortical recruitment during cell symmetry breaking or lateral branching was only observed for BNI-1.The earliest recruitment of BUD-6 and SPA-2 was observed during germ tube extension (Figures 1 and 2) [59]. Together with BNI-1, all three proteins localized to an apical cap, suggesting that a functional polarisome complex became constituted for the maintenance of polarized tip growth. The same applied for CAT homing during germling fusion (Figures 2 and 11), and extension of vegetative fusion hyphae in mature colonies (Figures 5 and 11). During lateral branching apical BUD-6 recruitment coincided with but did not precede Spk appearance (Figure 3C).

Together, this data suggests an exclusive role for BUD-6 in the maintenance of polarized tip growth in *N. crassa*, which contrasts with findings from budding yeast. Here punctate, cortical accumulations of Bud6-GFP have been observed prior to bud emergence [40]. However, the Aip3/Bud6 mutants of *S. cerevisiae* have not been found to be defective in the establishment of cytoskeletal polarity during budding. Loss over polarity control occurred later during bud enlargement, resulting in abnormally swollen cells. In fission yeast, Bud6 was found to localize to both cell tips and the cytokinetic ring [64]. Considering that Δ*bud6* cells of *S. pombe* had a specific defect in the efficient initiation of polarized growth indeed indicates an important role for Bud6 in the establishment of cell polarity, at least in fission yeast. In *A. nidulans* BudA-GFP has only been reported to accumulate at septation sites, but not at any other location associated with polarized growth [65]. Severe defects in polarity regulation in the *A. nidulans* Δ*budA* mutant, however, do suggest an important function for BudA in hyphal tip growth. In *C. albicans* and *A. gossypii* Bud6 has been localized to apical crescents/caps during tip growth maintenance [18], [66]; cortical recruitment prior to or during cell symmetry breaking has not been reported in these species.

Considered together, it appears that the role of BUD-6 in filamentous fungi is focused on the maintenance of cell polarity, whereas in yeasts BUD-6 also seems to operate during its establishment.

### Novel localization pattern of BUD-6 identified in the mature hyphal tip

Revealing was the finding that the apical localization pattern of BUD-6 in mature hyphal tips differed significantly from that of any other known polarisome component. This unexpected and so far unique localization pattern confirms the notion that the polarisome and Spk are distinct structures [66]. It furthermore suggests an entirely new function of the protein in the organization of the fungal tip growth apparatus. Absence from the Spk was surprising considering that the other two polarisome proteins partially (SPA-2, [59]) or fully (BNI-1) colocalized with this structure (Figure 12). To our knowledge, BUD-6 of *N. crassa* is the first Bud6 homolog localized as part of the apical growth apparatus in a filamentous fungus. It will be interesting to see if similar observations can be made in other filamentous fungal species.

### All three polarisome proteins are required to build a functional tip growth apparatus including the Spitzenkörper

We could show that deletion of *bud-6* or *bni-1* results in the complete absence of a detectable Spk (Figures 8 and 9). Although Δ*spa-2* did possess a Spk, its structure, however, was found to be distorted and accompanied by tip growth defects [59]. This demonstrates that all three polarisome components are required to establish and maintain the Spk, and furthermore to organise a functional tip growth apparatus. Other *N. crassa* mutants that fail to establish a Spk are the conventional kinesin-1 (*nkin-1*) mutant [67], [68], the exocyst component knock-out Δ*sec-5* [69], and the recently characterized GTPase module mutants Δ*cdc42*, Δ*rac-1* and Δ*cdc24* [46].

Similar links between Spk integrity and hyphal morphogenesis have been identified in *C. albicans* and *A. gossypii*. Deletion of either Ca*SPA2* or Ca*BUD6* resulted in germ tubes that were broader and less polarized than the wild type, and caused the regulatory light chain of Myosin 2 (Mlc1) to no longer localise to the Spk, but instead into a polarisome-like crescent [66]. A Spk-like accumulation of polarity components only existed in the hyphal growth form of this yeast. In all other cases, Mlc1 and Spa2, as well as FM4–64-stained vesicles, exclusively localized into an apical cap resembling the polarisome of pseudohyphae and yeast growth form of *C. albicans*. In *A. gossypii*, the accumulation of polarisome and exocyst components into an apical cap alone or an apical cap plus subtending Spk was associated with hyphal growth velocity, in that an increased extension rate of the tip coincided with the formation of a Spk [18]. Notably, very recently Spk-like vesicle accumulations have been identified at the tips of growing mating projections of *S. cerevisiae* and *C. albicans*, suggesting that more characteristics of hyphal tip growth have been conserved in yeasts than previously thought [70].

Our observation that during tip reorientation the apical BNI-1 clusters relocate before the Spk changes its position and the tip turns (Figure 12B), suggests that the formin exerts distinct functions in both structures to coordinate tip directionality. A possible scenario might include one population of BNI-1 that first initiates the nucleation of F-actin cables from the new tip location, before a second population of BNI-1 in the Spk core regulates the lateral displacement of the vesicle cluster towards the endpoint of the new cable track. Strains coexpressing BNI-1-GFP and the F-actin marker Lifeact-TagRFP-T [71] now provide excellent tools to study these dynamics in detail.

Taken together, these results show that polarisome components are essential to establish a functional Spk, and that their absence provokes changes in tip growth apparatus architecture and function which can significantly restrict morphogenesis. These findings also confirm that the presence of a vesicle supply centre is not *per se* required for polarized growth, but that its formation is essential for increased and targeted vesicle flow in order to support the hyphal tip shape and achieve fast extension speeds, and to coordinate tip directionality probably in interaction with the polarisome.

### BUD-6 and BNI-1 have polarisome-independent functions during septum formation and cytokinesis

It has been previously demonstrated that Spa2 homologs of *N. crassa* and *A. nidulans* are neither part of the landmarking machinery, nor components of the CAR driving septum constriction [59], [65]. Furthermore, we found that Δ*spa-2* strains of *N. crassa* were able to form septa and showed no defects in cytokinesis/conidiation (data not shown), clearly demonstrating that both processes occur independently of the central polarisome scaffolding protein. BUD-6 and BNI-1, on the other hand, were recruited to the incipient septation site before membrane invagination became visible by FM4–64 staining. During the actual septum formation process both proteins showed identical dynamics by remaining associated to the CAR. Whereas BNI-1 disappeared upon septum completion (Figure 13, and previously shown [62]), BUD-6 remained associated to the inner perimeter of the septal pore (Figure 1) for periods of about 4–5 hours and within a peripheral zone of about 1 mm behind the leading colony edge. This behavior indicates additional functions of the protein at this location. Key Rho signaling components, including RHO-1, RHO-2, RHO-3 and RHO-4, the associated GEFs BUD-3 and RGF-3, as well as the landmarking protein BUD-4, have been shown to exclusively function during septation, but not polarized tip growth [62], [72], [73]. Interestingly, most of these components also persist at septal pores, similar to BUD-6. Thus, it seems likely that these proteins interact at the septal pore, to allow rapid sealing in case physical injury occurs (see further discussion below). Interestingly, although a very similar phenotype for the *A. nidulans* Δ*budA* mutant has been reported [65], [74], in contrast to *N. crassa* Δ*bud-6*, Δ*budA* was able to form septa, indicating that BudA has only a minor role in septum formation in *Aspergillus*. Another difference seems to be that *A. nidulans* BudA did not remain at the pore for prolonged periods of time after septum completion.

A second polarisome-independent process is cytokinesis resulting in cell separation. In fungi this involves the formation of a primary cross wall, the inward growth of which is led by the CAR and results in two adjacent, cytologically separated cell compartments. Subsequently, additional layers of cell wall material are laid down at either side of the primary septum to form two secondary septa. Finally, the primary septum is dissolved by hydrolytic enzymes to allow physical cell separation, followed by removal of the septation machinery. In *N. crassa*, this sequence of events occurs during conidophore development and leads to the differentiation of asexual macroconidia [49]. During this process, BUD-6 and BNI-1 (but not SPA-2) are present in the CAR ultimately forming the primary septum, and contribute to the formation of secondary septa. In analogy to the formation of porous septa, BUD-6 remained associated with the cell poles upon completion of the secondary cross wall, whereas BNI-1 was only transiently present and did not last beyond the point when physical cell separation was achieved. A potential function of BUD-6 could involve recruitment of secretory vesicles containing cell wall material in order to strengthen the initially weak secondary septa. With progressing age of the colony, the number of conidia showing these accumulations decreased, suggesting that with maturation of the conidium, BUD-6 gradually dispersed from secondary septa and cell poles, respectively.

### Septal plugging, consolidation and repolarization

Hyphal wounding assays showed that immediately after the physical injury has been produced all three polarisome proteins rapidly localized to the septal plug, initiated by Woronin body recruitment [75]. Interestingly the three polarisome proteins exhibited slightly different dynamics: BUD-6, which was already present around the pore, became focused to the plug (Figure 4A); BNI-1 became newly recruited to an area around the pore, then focused to the plug (Figure 13A); and SPA-2 directly appeared at the plug (Figure 4D). This seemingly sequential occurrence might reflect a certain order of events during pore sealing. As neither Woronin bodies nor their key protein components are conserved in the Saccharomycotina [76], this probably polarisome-dependent function of BUD-6, SPA-2 and BNI-1 must be an ancient role removed during the evolution of the yeast forms from the filamentous fungal ancestor [77].

Complete pore sealing requires consolidation [78], [79], i.e. the establishment of a permanent seal through the accumulation of secretory vesicles depositing new cell wall material over the cytoplasmic side of the pore-occluding Woronin body, as well as plasma membrane resealing. Consolidation is usually followed by the reinitiation of polarized hyphal tip growth [80], [81]. The polarisome proteins are likely to be involved in the coordination of these processes, as all three components – the Woronin body, the polarisome proteins BUD-6, SPA-2 and BNI-1, and secretory vesicles – colocalized during this step (Figure 4). The notion that the Woronin body acts as an assembly platform for the polarity machinery, is supported by the fact that *N. crassa* mutants lacking the Woronin body core protein HEX-1 displayed a defect in the reinitiation of polarized growth [82]. The association between Woronin body and septum becomes established very early on during synthesis of the cross-wall [83], thus it would be interesting to investigate the spatiotemporal relationship between fluorescently labelled HEX-1 and polarisome proteins in greater detail.

Astonishing in these wounding assays was the speed at which a new hyphal tip regenerated from the sealed septum. Five to ten minutes after cutting the colony edge new growing tips were readily established (Figure 4), and sometimes several hyphal tips emerged from one sealed septum. Even more revealing were the quick changes in the apical localization patterns of the three polarisome proteins. Their spatial rearrangements occurred within the 10 minutes following septal plugging, and represent a ‘time-lapse’ of the morphogenetic transition from germling to mature hypha, which normally takes several hours. Thus, hyphal wounding assays provide an excellent model system to study the rearrangement of the tip growth apparatus in a time window of about 20 minutes, and can be studied with even greater spatiotemporal precision and less overall mycelial damage when applying laser dissection to selectively cut individual hyphae. This approach has provided the first evidence that components of the cell fusion machinery are involved in septal pore plugging and consolidation [84].

### Is the spatial location of cell fusion in the mature colony regulated by polarisome components?

All three polarisome proteins showed identical dynamics at the tips of CATs and fusion hyphae, terminating with their disappearance from the established fusion pore (Figures 2, 5 and 11). Interestingly, the fusion phenotype of the corresponding gene deletion mutants revealed some differences. Conidia of Δ*spa-2* only showed a slightly delayed onset of CAT-mediated cell fusion. However, in the end this mutant successfully established germling networks, and VHF in the mature colony was indistinguishable from the wild type [89]. Both, Δ*bni-1* and Δ*bud-6* mutants showed an intriguing phenotype in that VHF was established right at the leading edge of the colony (Figure 10); an unusual feature not seen in the wild type [53]. Due to the lack of conidia, germling fusion assays could not be performed in these two mutants. However, as evidence for VHF in the mature colonies of BUD-6 and BNI-1 deletion mutants has been found, and furthermore the presence of SPA-2 also was not essential for cell fusion, these data strongly suggest that a functional polarisome is dispensable for hyphal fusion but might assist in its regulation.

The peripheral fusion phenotype of Δ*bni-1* and Δ*bud-6* might be founded in the disruption of the apical tip growth apparatus. Apical dominance suppresses both the formation of branches at the leading edge of the mycelium [85], and prevents the development of fusion hyphae at the colony periphery [63]. Apical dominance was not clearly evident in the Δ*bni-1* and Δ*bud-6* mutants in which vegetative hyphae were much less differentiated and lacked distinct Spk. In both mutants this breakdown in apical dominance coincided with derepressed VHF at the colony edge. In the wild type, this repression of VHF could intriguingly be overcome in the presence of conidial germlings by some unknown mechanism. The physiological role of suppressed hyphal fusion must be the promotion of colony growth. Fusion at the leading edge would slow down colony extension and substrate exploration, and consequently lead to significantly delayed colony development that exactly matches the phenotypes of the two mutant mycelia.

Viable Bni1 null mutants have been generated in *A. gossypii* and *C. albicans*, and failed to form hyphae or develop abnormally swollen hyphae, respectively [86], [87]. A conditional SepA mutant of *A. nidulans* was found to be aseptate, exhibited an aberrant growth pattern and developed abnormally wide hyphae, indicating defects in targeted exocytosis [74], [88]. These morphogenetic alterations are in line with the severe phenotype of the Δ*bni-1* and Δ*bud-6* mutants of *N. crassa*.

In budding yeast it has been observed that all four polarisome components (Spa2, Pea2, Aip3/Bud6 and Bni1), as well as the cell fusion proteins Fus1 and Fus2 are required to terminate mating projection growth prior to cell fusion (reviewed in [35]). Likewise, the termination of polarized tip growth of CATs and vegetative fusion hyphae is a prerequisite for successful cell fusion. A shared phenotype amongst fusion mutants of *N. crassa* is continued tip growth of fusion hyphae upon physical contact with other hyphae [89], [90]. *Spa2*Δ cells of budding yeast showed a mating cell fusion defect similar to *fus1*Δ and *fus2*Δ [91]. To our knowledge, whether or not Bud6 is required for mating cell fusion has so far not been reported in the yeast literature. Fus1 has been found to regulate the opening and expansion of the fusion pore between mating yeasts [92]. As Fus orthologs do not exist in *N. crassa* it is tempting to speculate that some polarisome components might have this role in filamentous fungi. Clearly, more detailed functional studies are required to test this idea.

### The complex filamentous fungal polarisome

In summary, this investigation has shown that filamentous fungal polarisome components show higher functional diversity than their homologs in yeasts. To elucidate further molecular details of the filamentous fungal polarisome it appears reasonable to focus the attention on polarisome-dependent processes, i.e. exclude septation and cytokinesis in this context. The complex arrangement of the polarisome in mature hyphal tips, comprising the subapical BUD-6 cloud, BNI-1 clusters within the Spk core and the apical cap, and SPA-2 partially overlapping with the Spk but also fanning outwards into the apical dome, is a novel architectural feature of the filamentous tip growth apparatus that deserves more detailed investigation. Alongside the search for novel components of the filamentous fungal polarisome, understanding the individual functions of the three known proteins in greater detail, and especially in interaction with the actin cytoskeleton, poses interesting challenges for the future.

## Materials and Methods

### Strains and culture conditions

Strains of *Neurospora crassa* used in this study are listed in Table 2. Unless otherwise stated, strains were cultured at 28°C on Vogel's Minimal Medium (VMM) [47] supplemented with 2% sucrose and solidified with 1.5% agar when needed. The auxotrophic Δ*mus51* strain was grown on VMM agar supplemented with 0.5 mg/ml histidine. All experimental manipulations were according to standard techniques [48], [49]. Preparation of conidial cell suspensions for the assessment of germling development and observation of CAT-mediated cell fusion were performed as described previously [26], [50], [51].

### Homokaryon purification using crossing

Homokaryotic Δ*bud-6* strains were generated by selecting ascospores obtained from a sexual cross between the heterokaryotic Δ*bud-6* mat a gene deletion strain and Δ*sad-2* mat A (Table 2) on hygromycin B medium (200 µg/ml), and evaluating the percentage of co-segregation of the mutant phenotype with hygromycin resistance. Absence of the native *bud-6* locus from the genome of isolated Δ*bud-6* mutants was verified by PCR (Figure S1).

### Homokaryon purification using conidida

The heterokaryotic Δ*bud-6* or Δ*bni-1* strains were grown on VMM containing 200 µg/ml hygromycin B until enough asexual spores (macro- and microconidia) have developed. Conidia were harvested in 1 ml sterile dH_2_O and adjusted by eye to yield a slightly cloudy, orange suspension. 150 µl of a 1∶5000 diluted spore suspension were evenly distributed on fresh VMM supplemented with 200 µg/ml hygromycin B using glass beads, and subsequently incubated at 30°C overnight. Developing microcolonies derived from single conidia and showing a mutant phenotype were excised under the stereomicroscope using a sterile syringe needle, and transferred onto small (5 cm diameter) Petri dishes containing VMM with hygromycin B. These were incubated at 30°C, and colony development assessed over the following days. In case wt-like colonies developed, the procedure was repeated three times or until prominent phenotype changes occurred. Subsequently genomic DNA was extracted from selected clones and changes in the genome were verified by multiplex PCR as explained in detail elsewhere [52].

### Construction of pMY1 and pMY2

The *Neurospora crassa* locus NCU08468.3, was identified by BLASTp analysis in the Broad Institute Genome database (http://www.broadinstitute.org/scientific-community/data) as the sole homolog of the *S. cerevisae* Aip3/Bud6 locus SCRG04267.1. NCU08468.3 encodes a protein of 1001 bp length with an annotated actin-interaction protein domain. In the most recent annotation of the *bud-6* locus (NCU08468.5) an additional 49 amino acid-encoding N-terminal extension has been identified, which however, is not conserved in the orthologous budding yeast locus, and the resulting gene product does not represent any specific protein domain. Extraction of genomic DNA from the *N. crassa* strain N150 (FGSC #9013) was carried out using a DNeasy Plant Extraction Kit (Qiagen, Inc.) For this, mycelium from a 48 h VMM liquid culture (28°C, 250 rpm, dark) was filtered and pulverized in liquid nitrogen, and processed through the protocol according to manufacturer's recommendations. Standard PCR and cloning procedures [53] were used to generate carboxyterminal BUD-6-GFP and BUD-6-mCherry fusion proteins. The *bud-6* gene was amplified by PCR from genomic DNA extracted from the *N. crassa* wild type strain N150 (FGSC #9013), using oligonucleotides Bud6-XbaI-F 5′-GCTCTAGAATGGGTCCCCAAGCTGGCAT-3′ and Bud6-PacI-R 5′-CCTTAATTAACTCTTCCTCCTCTTCCTCC-3′. The PCR reaction was performed in a NYX Technik Amplitronix A6 (ATC401 Apollo®) Thermal Cycler with Platinum® Taq High Fidelity DNA polymerase (Invitrogen) according to the manufacturer's instructions. The gel-purified PCR product was digested with *Xba*I and *PacI* and subsequently ligated into accordingly linearized pMF272 [54] and pJV15-2 [55], respectively. In-frame integration of the BUD-6 encoding fragment in the resulting vectors pMY1 and pMY2 was verified by restriction digest analysis and sequencing. Ectopic expression of the fusion protein was under control of the glucose-repressible *ccg-1* promoter which ensures low level expression on 2% sucrose-supplemented minimal medium [54], [56].

### Transformation and transformant selection

Transformations were performed using a standard electroporation protocol for *N. crassa* [57]. To generate strains expressing BUD-6-GFP and BUD-6-mCherry, linearized (*Nde*I-digested) pMY1 and pMY2, respectively, were targeted to the *his-3* locus of strain FGSC 9717 (*mat A his-3^−^ Δmus-51*::*bar^+^*). Transformants were selected by recovery of histidine auxotrophy on selection medium, and screened for signal intensity and localization using laser scanning confocal microscopy (LSCM). From all selected transformants five BUD-6-GFP expressing strains and one BUD-6-mCherry expressing strain displayed fluorescence signals suitable for subsequent live-cell imaging studies. Selected BUD-6-GFP and BUD-6-mCHFP transformants were named NECL46 and NECL47, respectively (Table 2). As the fluorescence signal of the mCherry fusion protein was considerably weaker and prone to rapid photobleaching, compared to the equivalent GFP fusion construct, most experiments were conducted with strain NECL46 (Table 2). Visualization of BNI-1 dynamics was performed using the complemented Δ*bni-1*(BNI-1-GFP) strain kindly provided by Justa-Schuch et al. (2010).

### Fluorescent staining

To visualize the plasma membrane and organelle membranes, mature hyphae and germlings were stained with 2 µM of the lipophilic marker dye FM4–64 (prepared from 200 µM stock in DMSO; Molecular Probes, Eugene, OR) as previously described in detail [58].

### Live-cell imaging microscopy

For live-cell imaging of mature hyphae, mycelium incubated overnight on solid VMM agar was prepared using the “inverted agar block” method [58] and imaged on an inverted LSCM (LSM 510 Meta, Carl Zeiss, Göttingen, Germany), using 63×/1.4 NA Plan-Apochromat and 100×/1.3 NA Plan-Neofluar oil immersion objectives. Fluorescence signals were detected with the following settings: GFP (excitation 488 nm from an Argon laser, 545 nm dichroic mirror, emission 505–550 nm), mCherry (excitation 543 nm from an HeNe laser, 570 nm dichroic mirror, emission 574–691 nm) and FM4–64 (excitation 488 nm from an Argon laser, 570 nm dichroic mirror, emission 574–691 nm). Digital images were captured and analyzed using the implemented LSM 510 software (version 3.2; Carl Zeiss). A transmitted light detector permitted simultaneous recording of widefield phase contrast images with confocal fluorescence signals. Additionally, for the acquisition of some 3D time series a DeltaVision epifluorescence microscope (Applied Precision, Issaquah, WA) was used, and germlings prepared as described previously [26]. Final image and time series manipulation was performed using the ImageJ platform (http://rsbweb.nih.gov/ij/).

## Supporting Information

Figure S1 **Genetic verification of gene deletion mutants by PCR.** (**A**) Colony PCR results from two isolated clones (NECL48-5 and NECL48-8, Table 2) confirming absence of the 3.2 kb fragment amplified from the *bud-6* ORF in the wild type. The bands <500 bp are likely to be unspecific products of the used oligonucleotides, as they show up equally in all three strains. (**B**) Multiplex PCR genotyping results confirming that through isolation of monosporic microcolonies wild type nuclei carrying the *bni-1* gene have been removed from the heterokaryotic Δ*bni-1* strain (FGSC 11490, Δ*mus51* background), generating the homokaryon selected (HS) Δ*bni-1* strain. The wild type control (left panel) contains all gene loci except the KO cassette targeted to the *bni-1* locus. The 700 bp fragment at the bottom of each lane was amplified from the actin locus and served as an internal reaction control for each individual PCR, particularly important for those reactions where no other product is expected due to the absence of the tested locus. The heterokaryotic gene deletion strain (middle panel) still contains the native *bni-1* gene, but also a population of transformed nuclei harboring the KO cassette at this locus. As Δ*mus-51* strains were used as recipients of the KO cassette, the *mus-51* gene is absent from any Δ*bni-1* gene deletion strain that has not been backcrossed to a wild type. In the vegetatively selected Δ*bni-1* homokaryon (right panel) the primer pairs detecting the 5′ region of the *bni-1* locus and parts of this ORF anywhere in the genome did not result any product, confirming complete absence of this locus from the genome in the selected mutant strains.
(TIF)

Figure S2 **Ectopic expression of fluorescent BUD-6 fusion constructs did not interfere with colony development.** (**A**) Colony morphology of wild type N150, NECL46-9 and NECL47-5 after 24 and 48 hours of growth on Vogel's medium at 28°C. No differences with respect to general colony architecture or conidiation pattern were observed between wild type and transformants. (**B**) Colony extension rates were measured every two hours over a period of 24 h and statistically analyzed (**C**). No significant differences between the three strains could be observed.
(TIF)

Figure S3 **Changes in the abundance of BUD-6 clusters in developing conidia.** VMM slants were inoculated with the BUD-6-GFP expressing strain NECL46-9 (Table 2), and continuously incubated for 10 days at 28°C. At the indicated time points conidial samples were taken and observed using laser confocal microscopy. As this analysis was based on sampling only single random optical sections of fields of conidia the results are an underestimate of the number of clusters present (i.e. clusters outside the focal plane were not captured). Each optical section was taken at a random plane through the spores, and thus provides the average distribution pattern within the cell population at each time point. (**A**) Example image showing the scored pattern of BUD-6 cluster distribution in freshly harvested conidia. Scale bar, 10 µm. (**B**) The graph displays the percentage of cells showing the particular BUD-6 cluster distribution pattern at the indicated sampling times hours post inoculation (hpi). With colony development the number of conidia with cortical clusters at one or both cell poles decreases, whereas the number of cells containing intracellular clusters increases. Together, this data indicates that the changes in BUD-6 cluster distribution are probably connected to conidial maturation, which causes the redistribution of BUD-6 from sites of cytokinesis to internal compartments.
(TIF)

Movie S1 Recruitment of BUD-6-GFP to the incipient site of CAR assembly occurs seconds before plasma membrane invagination becomes visible by FM4–64 staining. BUD-6-GFP remains associated to the CAR during septum formation.
(MP4)

Movie S2 BUD-6-GFP accumulates at homing CAT tips and remains present at the fusion site until cytoplasmic continuity is established. Note the bright clusters of BUD-6 at other locations along the cell cortex, and the disappearing ring of BUD-6 at the fusion site.
(MP4)

Movie S3 SPA-2-GFP accumulates at homing CAT tips and remains present at the fusion site until cytoplasmic continuity is established. During CAT homing and fusion germ tube growth is arrested, but resumes shortly after fusion pore opening.
(MP4)

Movie S4 BUD-6-GFP forms a highly dynamic subapical cloud surrounding the Spitzenkörper (stained with FM4–64). The merged seqeunce shows that BUD-6-GFP does not colocalize with the Spitzenkörper.
(MP4)

Movie S5 Time course of concatenated z-stacks showing BUD-6-GFP recruitment to the septal plug prior to tip repolarization from the sealed septum. BUD-6 and membranous material (FM4–64 staining) accumulate around the Woronin body (phase contrast).
(MP4)

Movie S6 Time course of concatenated z-stacks showing BUD-6-GFP recruitment during tip repolarization from the sealed septum. BUD-6 accumulations are present left and right to the septal plug even before the new tip emerges. An apical BUD-6 crescent accumulates as soon as elongation growth of the tip commences.
(MP4)

Movie S7 Time course of concatenated z-stacks showing SPA-2-GFP recruitment during tip repolarization from the sealed septum. SPA-2-GFP becomes recruited to the septal plug and permanently remains associated to the emerging and elongating tip as apical cap.
(MP4)

Movie S8 BUD-6-GFP recruitment to tips of vegetative fusion hyphae and the rim of the opening fusion pore. Shortly after cytoplasmic continuity between the two hyphae is established, BUD-6-GFP fluorescence disappears from the fusion site.
(MP4)

Movie S9 Vegetative hyphal fusion at the leading colony edge of Δ*bni-1*. Due to the lack of septa, huge vacuoles accumulate at the edge of the mutant colony, which can be seen passing through fusion pores.
(MP4)

Movie S10 A group of germlings demonstrating dynamics of BN-1-GFP recruitment during different stages of cell fusion. Taken together, this sequence demonstrates that BNI-1 is present at homing CAT tips, focuses at the attachment site, forms an opening ring of fluorescence during fusion pore formation, and finally disappears from the fusion site. Note, the cell in the middle becomes lifted upwards due to the torque forces transmitted upon cell-cell attachment, and thus disappears from the focal plane. This “spore-torque” is commonly observed when imaging germling fusion of *N. crassa* in liquid medium.
(MP4)

Movie S11 Time course of concatenated z-stacks showing BNI-1-GFP recruitment during tip repolarization from the sealed septum. BNI-1-GFP accumulates at the septal plug,, then organizes into an apical cap as the new tip emerges. Shortly after, as the length of the new hyphae (the first one emerging in the middle) increases, BNI-1-GPF reorganizes into apical cap with subtending apical spot, indicating beginning formation of a Spitzenkörper.
(MP4)

## References

[1] Piel M, Tran PT (2009). Cell shape and cell division in fission yeast.. Current Biology.

[2] Pollard TD, Wu JQ (2010). Understanding cytokinesis: lessons from fission yeast.. Nature Reviews Molecular Cell Biology.

[3] Bornens M (2008). Organelle positioning and cell polarity.. Nature Reviews Molecular and Cellular Biolology.

[4] Li R, Gundersen GG (2008). Beyond polymer polarity: how the cytoskeleton builds a polarized cell.. Nature Reviews Molecular Cell Biology.

[5] Perez P, Rincon SA (2010). Rho GTPases: regulation of cell polarity and growth in yeasts.. Biochemical Journal.

[6] Slaughter BD, Smith SE, Li R (2009). Symmetry breaking in the life cycle of the budding yeast.. Cold Spring Harbor Perspectives in Biology.

[7] Wedlich-Söldner R, Li R (2008). Yeast and fungal morphogenesis from an evolutionary perspective.. Seminars in Cell and Developmental Biology.

[8] Harris SD (2006). Cell polarity in filamentous fungi: shaping the mold.. International Review of Cytology.

[9] Harris SD (2008). Branching of fungal hyphae: regulation, mechanisms and comparison with other branching systems.. Mycologia.

[10] Harris SD, Momany M (2004). Polarity in filamentous fungi: moving beyond the yeast paradigm.. Fungal Genetics and Biology.

[11] Roca GM, Arlt J, Jeffree CE, Read ND (2005). Cell biology of conidial anastomosis tubes in *Neurospora crassa*.. Eukaryotic Cell.

[12] Roca GM, Read ND, Wheals AE (2005). Conidial anastomosis tubes in filamentous fungi.. FEMS Microbiological Letters.

[13] Read ND, Lichius A, Shoji J, Goryachev AB (2009). Self-signalling and self-fusion in filamentous fungi.. Current Opinion in Microbiology.

[14] Trinci APJ (1973). Growth of wild-type and spreading colonial mutants of *Neurospora crassa* in batch culture and on agar medium.. Archives of Microbiology.

[15] Momany M (2002). Polarity in filamentous fungi: establishment, maintenance and new axes.. Current Opinion in Microbiology.

[16] Read ND, Fleißner A, Roca MG, Glass NL, Borkovich KA, Ebbole D (2010). Hyphal fusion.. Cellular and Molecular Biology of Filamentous Fungi.

[17] Jones LA, Sudbery PE (2010). Spitzenkörper, exocyst, and polarisome components in *Candida albicans* hyphae show different patterns of localization and have distinct dynamic properties.. Eukaryotic Cell.

[18] Köhli M, Galati V, Boudier K, Roberson RW, Philippsen P (2008). Growth-speed-correlated localization of exocyst and polarisome components in growth zones of *Ashbya gossypii* hyphal tips.. Journal of Cell Science.

[19] Harris SD (2009). The Spitzenkörper: a signalling hub for the control of fungal development?. Molecular Microbiology.

[20] Sudbery P (2011). Fluorescent proteins illuminate the structure and function of the hyphal tip apparatus.. Fungal Genetics and Biology.

[21] Lichius A, Berepiki A, Read ND (2011). Form follows function: the versatile fungal cytoskeleton.. Fungal Biology.

[22] Berepiki A, Lichius A, Read ND (2011). Actin organization and dynamics in filamentous fungi.. Nature Reviews Microbiology.

[23] Taheri-Talesh N, Horio T, Araujo-Bazán L, Dou X, Espeso EA (2008). The tip growth apparatus of *Aspergillus nidulans*.. Molecular Biology of the Cell.

[24] Upadhyay S, Shaw BD (2008). The role of actin, fimbrin and endocytosis in growth of hyphae in *Aspergillus nidulans*.. Molecular Microbiology.

[25] Araujo-Bazán L, Penalva MA, Espeso EA (2008). Preferential localization of the endocytic internalization machinery to hyphal tips underlies polarization of the actin cytoskeleton in *Aspergillus nidulans*.. Molecular Microbiology.

[26] Berepiki A, Lichius A, Shoji J, Tilsner J, Read ND (2010). F-actin dynamics in *Neurospora crassa*.. Eukaryotic Cell.

[27] Delgado-Álvarez DL, Callejas-Negrete OA, Gómez N, Freitag M, Roberson RW (2010). Visualization of F-actin localization and dynamics with live cell markers in *Neurospora crassa*.. Fungal Genetics and Biology.

[28] Shaw BD, Chung DW, Wang CL, Quintanilla LA, Upadhyay S (2011). A role for endocytic recycling in hyphal growth.. Fungal Biology.

[29] Sheu YJ, Santos B, Fortin N, Costigan C, Snyder M (1998). Spa2p interacts with cell polarity proteins and signaling components involved in yeast cell morphogenesis.. Molecular and Cellular Biology.

[30] Evangelista M, Blundell K, Longtine MS, Chow CJ, Adames N (1997). Bni1p, a yeast formin linking cdc42p and the actin cytoskeleton during polarized morphogenesis.. Science.

[31] Fujiwara T, Tanaka K, Mino A, Kikyo M, Takahashi K (1998). Rho1p-Bni1p-Spa2p interactions: implication in localization of Bni1p at the bud site and regulation of the actin cytoskeleton in *Saccharomyces cerevisiae*.. Molecular Biology of the Cell.

[32] Moseley JB, Sagot I, Manning AL, Xu Y, Eck MJ (2004). A conserved mechanism for Bni1- and mDia1-induced actin assembly and dual regulation of Bni1 by Bud6 and profilin.. Molecular Biology of the Cell.

[33] Sagot I, Klee SK, Pellman D (2002). Yeast formins regulate cell polarity by controlling the assembly of actin cables.. Nature Cell Biology.

[34] Ozaki-Kuroda K, Yamamoto Y, Nohara H, Kinoshita T, Fujiwara T (2011). Dynamic localization and function of Bni1p at the sites of directed growth in *Saccharomyces cerevisiae*.. Molecular and Cellular Biology.

[35] Bidlingmaier S, Snyder M (2004). Regulation of polarized growth initiation and termination cycles by the polarisome and Cdc42 regulators.. Journal of Cell Biology.

[36] van Drogen F, Peter M (2002). Spa2p functions as a scaffold-like protein to recruit the Mpk1p MAP kinase module to sites of polarized growth.. Current Biology.

[37] Tcheperegine SE, Gao XD, Bi E (2005). Regulation of cell polarity by interactions of Msb3 and Msb4 with Cdc42 and polarisome components.. Molecular and Cellular Biology.

[38] Chenevert J, Valtz N, Herskowitz I (1994). Identification of genes required for normal pheromone-induced cell polarization in *Saccharomyces cerevisiae*.. Genetics.

[39] Valtz N, Herskowitz I (1996). Pea2 protein of yeast is localized to sites of polarized growth and is required for efficient mating and bipolar budding.. Journal of Cell Biology.

[40] Amberg DC, Zahner JE, Mulholland JW, Pringle JR, Botstein D (1997). Aip3p/Bud6p, a yeast actin-interacting protein that is involved in morphogenesis and the selection of bipolar budding sites.. Molecular Biology of the Cell.

[41] Moseley JB, Goode BL (2005). Differential activities and regulation of *Saccharomyces cerevisiae* formin proteins Bni1 and Bnr1 by Bud6.. Journal of Biological Chemistry.

[42] Delgehyr N, Lopes CS, Moir CA, Huisman SM, Segal M (2008). Dissecting the involvement of formins in Bud6p-mediated cortical capture of microtubules in *S. cerevisiae*.. Journal of Cell Science.

[43] Butty AC, Perrinjaquet N, Petit A, Jaquenoud M, Segall JE (2002). A positive feedback loop stabilizes the guanine-nucleotide exchange factor Cdc24 at sites of polarization.. EMBO Journal.

[44] Goryachev AB, Pokhilko AV (2008). Dynamics of Cdc42 network embodies a turing-type mechanism of yeast cell polarity.. FEBS letters.

[45] Knechtle PJ, Dietrich F, Philippsen P (2003). Maximal polar growth potential depends on the polarisome component AgSpa2 in the filamentous fungus *Ashbya gossypii*.. Mol Biol Cell.

[46] Araujo-Palomares CL, Richthammer C, Seiler S, Castro-Longoria E (2011). Functional characterization and cellular dynamics of the CDC42-RAC-CDC-24 module in *Neurospora crassa*.. PLoS One.

[47] Vogel HJ (1956). A convenient growth medium for *Neurospora* (Medium N).. Microbiology and Genetics Bulletin.

[48] Davis RH, Perkins DD (2002). *Neurospora*: a model of model microbes.. Nature Reviews Genetics.

[49] Davis RH (2000). *Neurospora*: contributions of a model organism.

[50] Lichius A, Roca MG, Read ND (2010). How to distinguish conidial anastomosis tubes (CATs) from germ tubes.. The *Neurospora* protocol guide.

[51] Roca MG, Lichius A, Read ND (2010). How to analyze and quantify conidial anastomosis tube (CAT)-mediated cell fusion.. The *Neurospora* protocol guide.

[52] Lichius A, Lord KM, Jeffree CE, Oborny R, Boonyarungsrit P (2011). Protoperithecial morphogenesis in *Neurospora crassa* and the role of MAPK signaling in this process.. Eukaryotic Cell.

[53] Sambrook J, Russell DW (2001). Molecular Cloning: A laboratory manual.

[54] Freitag M, Hickey PC, Raju NB, Selker EU, Read ND (2004). GFP as a tool to analyze the organization, dynamics and function of nuclei and microtubules in *Neurospora crassa*.. Fungal Genetics and Biology.

[55] Verdín J, Bartnicki-Garcia S, Riquelme M (2009). Functional stratification of the Spitzenkörper of *Neurospora crassa*.. Molecular Microbiology.

[56] McNally MT, Free SJ (1988). Isolation and characterization of a *Neurospora* glucose-repressible gene.. Current Genetics.

[57] Margolin BS, Freitag M, Selker EU (1997). Improved plasmids for gene targeting at the *his-3* locus of *Neurospora crassa* by electroporation.. Fungal Genetics Newsletter.

[58] Hickey PC, Swift SR, Roca MG, Read ND (2005). Live-cell Imaging of filamentous fungi using vital fluorescent dyes and confocal microscopy.. Methods in Microbiology: Elsevier B. V.

[59] Araujo-Palomares CL, Riquelme M, Castro-Longoria E (2009). The polarisome component SPA-2 localizes at the apex of *Neurospora crassa* and partially colocalizes with the Spitzenkörper.. Fungal Genetics and Biology.

[60] Ramos-García SL, Roberson RW, Freitag M, Bartnicki-Garcia S, Mourino-Pérez RR (2009). Cytoplasmic bulk flow propels nuclei in mature hyphae of *Neurospora crassa*.. Eukaryotic Cell.

[61] Riquelme M, Yarden O, Bartnicki-Garcia S, Bowman B, Castro-Longoria E (2011). Architecture and development of the *Neurospora crassa* hypha – a model cell for polarized growth.. Fungal Biology.

[62] Justa-Schuch D, Heilig Y, Richthammer C, Seiler S (2010). Septum formation is regulated by the RHO4-specific exchange factors BUD3 and RGF3 and by the landmark protein BUD4 in *Neurospora crassa*.. Molecular Microbiology.

[63] Hickey PC, Jacobson DJ, Read ND, Louise Glass N (2002). Live-cell imaging of vegetative hyphal fusion in *Neurospora crassa*.. Fungal Genetics and Biology.

[64] Glynn JM, Lustig RJ, Berlin A, Chang F (2001). Role of bud6p and tea1p in the interaction between actin and microtubules for the establishment of cell polarity in fission yeast.. Current Biology.

[65] Virag A, Harris SD (2006). Functional characterization of *Aspergillus nidulans* homologues of *Saccharomyces cerevisiae* Spa2 and Bud6.. Eukaryotic Cell.

[66] Crampin H, Finley K, Gerami-Nejad M, Court H, Gale C (2005). *Candida albicans* hyphae have a Spitzenkörper that is distinct from the polarisome found in yeast and pseudohyphae.. Journal of Cell Science.

[67] Riquelme M, Yarden O, Bartnicki-Garcia S, Bowman B, Castro-Longoria E (2011). Architecture and development of the *Neurospora crassa* hypha - a model cell for polarized growth.. Fungal Biology.

[68] Seiler S, Plamann M (2003). The genetic basis of cellular morphogenesis in the filamentous fungus *Neurospora crassa*.. Molecular Biology of the Cell.

[69] Beltrán-Aguilar A (2006). Caracterización macroscópica y microscópica de la mutante de *Neurospora crassa* sec5.

[70] Chapa YLB, Lee S, Regan H, Sudbery P (2011). The mating projections of *Saccharomyces cerevisiae* and *Candida albicans* show key characteristics of hyphal growth.. Fungal Biology.

[71] Lichius A, Read ND (2010). A versatile set of Lifeact-RFP expression plasmids for live-cell imaging of F-actin in filamentous fungi.. Fungal Genetics Report.

[72] Rasmussen CG, Glass NL (2007). Localization of RHO-4 indicates differential regulation of conidial versus vegetative septation in the filamentous fungus *Neurospora crassa*.. Eukaryotic Cell.

[73] Rasmussen CG, Glass NL (2005). A rho-type GTPase, *rho-4*, is required for septation in *Neurospora crassa*.. Eukaryotic Cell.

[74] Sharpless KE, Harris SD (2002). Functional characterization and localization of the *Aspergillus nidulans* formin SEPA.. Molecular Biology of the Cell.

[75] Trinci APJ, Collinge AJ (1973). Occlusion of septal pores of damaged hyphae of *Neurospora crassa* by hexagonal crystals.. Protoplasma.

[76] Jedd G (2011). Fungal evo-devo: organelles and multicellular complexity.. Trends in Cell Biology.

[77] Liu YJ, Hall BD (2004). Body plan evolution of ascomycetes, as inferred from an RNA polymerase II phylogeny.. Proc Natl Acad Sci U S A.

[78] Collinge AJ, Markham P (1987). Response of severed *Penicillium chrysogenum* hyphae following rapid Woronin body plugging of septal pores.. FEMS Microbiology Letters.

[79] Collinge AJ, Markham P (1985). Woronin bodies rapidly plug septal pores of severed *Penicillium chrysogenum* hyphae.. Experimental Mycology.

[80] Buller AHR (1933). Researches on Fungi.

[81] Markham P (1994). Occlusion of septal pores in filamentous fungi.. Mycological Research.

[82] Jedd G, Chua NH (2000). A new self-assembled peroxisomal vesicle required for efficient resealing of the plasma membrane.. Nature Cell Biology.

[83] Markham P, Coollinge AJ (1987). Woronin bodies of filamentous fungi.. FEMS Microbiology Review.

[84] Fleißner A, Glass NL (2007). SO, a protein involved in hyphal fusion in *Neurospora crassa*, localizes to septal plugs.. Eukaryotic Cell.

[85] Rayner ADM (1991). The challenge of the individualistic mycelium.. Mycologia.

[86] Martin R, Walther A, Wendland J (2005). Ras1-induced hyphal development in *Candida albicans* requires the formin Bni1.. Eukaryotic Cell.

[87] Schmitz HP, Kaufmann A, Kohli M, Laissue PP, Philippsen P (2006). From function to shape: a novel role of a formin in morphogenesis of the fungus *Ashbya gossypii*.. Molecular Biology of the Cell.

[88] Harris SD, Hamer L, Sharpless KE, Hamer JE (1997). The *Aspergillus nidulans* sepA gene encodes an FH1/2 protein involved in cytokinesis and the maintenance of cellular polarity.. EMBO Journal.

[89] Lichius A (2010). Cell Fusion in *Neurospora crassa*.

[90] Fleißner A, Sarkar S, Jacobson DJ, Roca GM, Read ND (2005). The *so* locus is required for vegetative cell fusion and postfertilization events in *Neurospora crassa*.. Eukaryotic Cell.

[91] Gammie AE, Brizzio V, Rose MD (1998). Distinct morphological phenotypes of cell fusion mutants.. Molecular Biology of the Cell.

[92] Nolan S, Cowan AE, Koppel DE, Jin H, Grote E (2006). FUS1 Regulates the Opening and Expansion of Fusion Pores between Mating Yeast.. Molecular Biology of the Cell.

[93] Carbo N, Perez-Martin J (2008). Spa2 is required for morphogenesis but it is dispensable for pathogenicity in the phytopathogenic fungus *Ustilago maydis*.. Fungal Genetics and Biology.

[94] Meyer V, Arentshorst M, van den Hondel CA, Ram AF (2008). The polarisome component SpaA localises to hyphal tips of *Aspergillus niger* and is important for polar growth.. Fungal Genetics and Biology.

[95] Leeder AC, Turner G (2008). Characterisation of *Aspergillus nidulans* polarisome component BemA.. Fungal Genetics and Biology.

