# Supplementary figures and images for: Comparative Live-Cell Imaging Analyses of SPA-2, BUD-6 and BNI-1 in Neurospora crassa Reveal Novel Features of the Filamentous Fungal Polarisome
Source: PLoS One. 2012 Jan 24;7(1):e30372. doi: 10.1371/journal.pone.0030372 (PMC3265482; doi:10.1371/journal.pone.0030372)

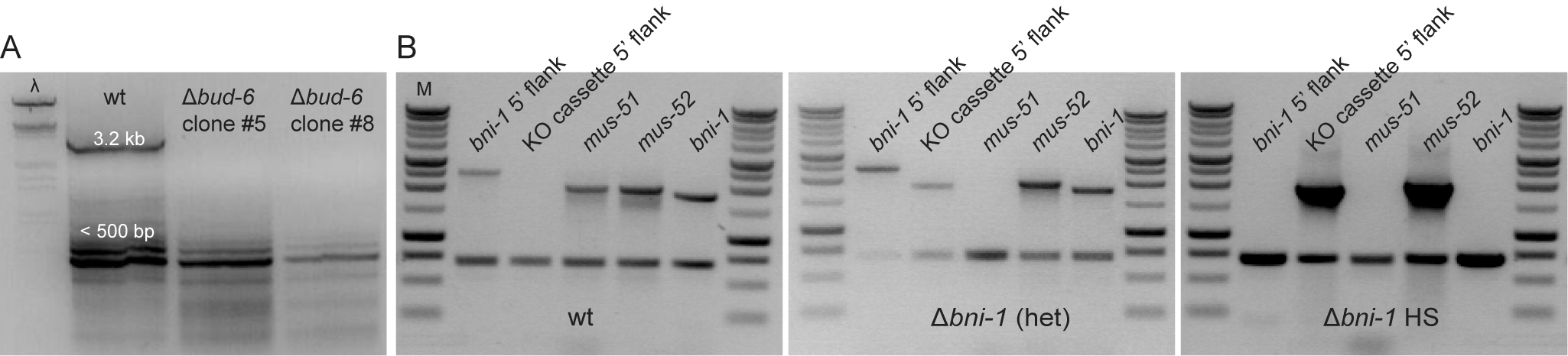

Supplement: Figure S1 — Genetic verification of gene deletion mutants by PCR. (A) Colony PCR results from two isolated clones (NECL48-5 and NECL48-8, Table 2) confirming absence of the 3.2 kb fragment amplified from the bud-6 ORF in the wild type. The bands <500 bp are likely to be unspecific products of the used oligonucleotides, as they show up equally in all three strains. (B) Multiplex PCR genotyping results confirming that through isolation of monosporic microcolonies wild type nuclei carrying the bni-1 gene have been removed from the heterokaryotic Δbni-1 strain (FGSC 11490, Δmus51 background), generating the homokaryon selected (HS) Δbni-1 strain. The wild type control (left panel) contains all gene loci except the KO cassette targeted to the bni-1 locus. The 700 bp fragment at the bottom of each lane was amplified from the actin locus and served as an internal reaction control for each individual PCR, particularly important for those reactions where no other product is expected due to the absence of the tested locus. The heterokaryotic gene deletion strain (middle panel) still contains the native bni-1 gene, but also a population of transformed nuclei harboring the KO cassette at this locus. As Δmus-51 strains were used as recipients of the KO cassette, the mus-51 gene is absent from any Δbni-1 gene deletion strain that has not been backcrossed to a wild type. In the vegetatively selected Δbni-1 homokaryon (right panel) the primer pairs detecting the 5′ region of the bni-1 locus and parts of this ORF anywhere in the genome did not result any product, confirming complete absence of this locus from the genome in the selected mutant strains. (TIF) [file pone.0030372.s001.tif]

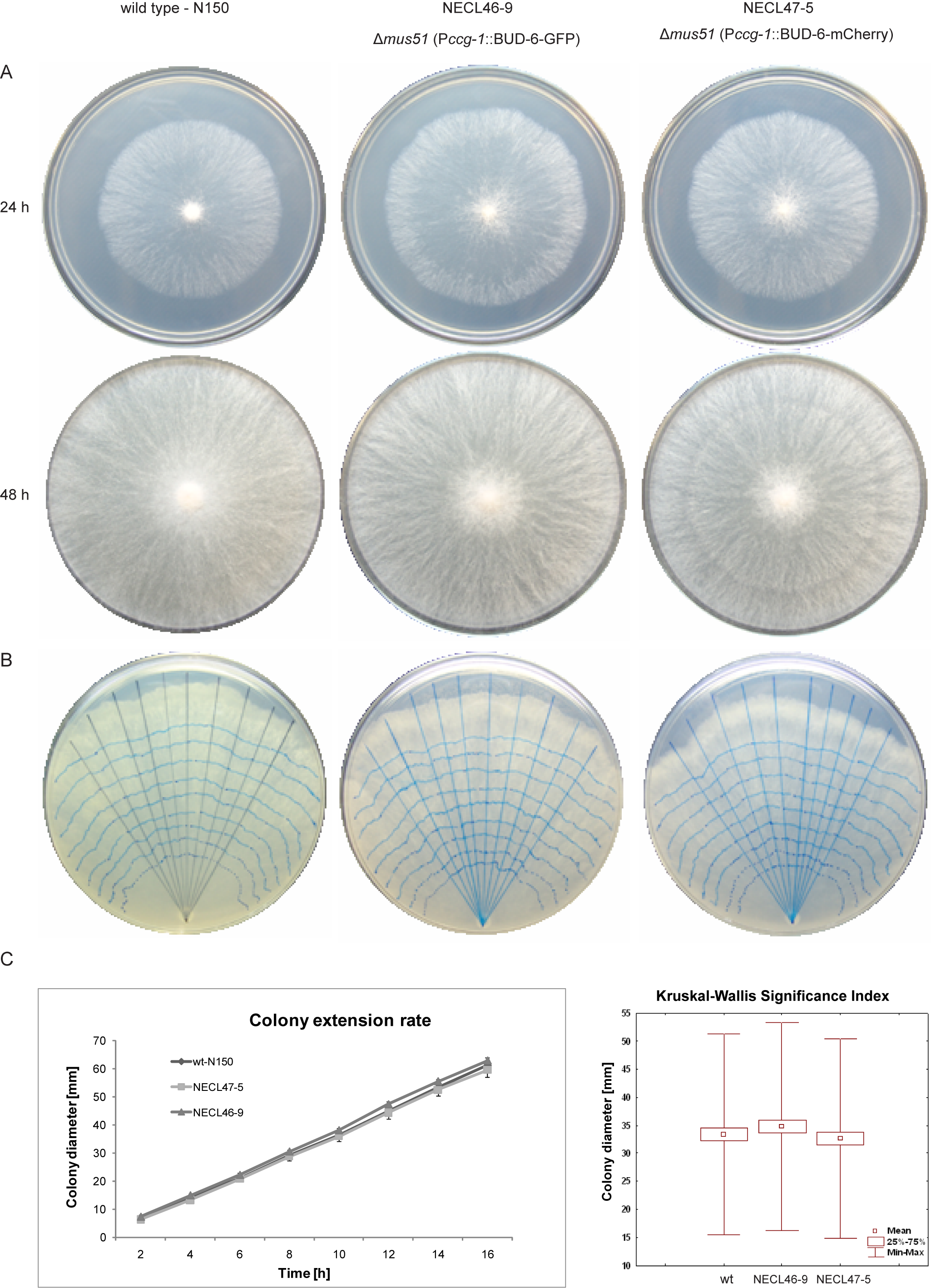

Supplement: Figure S2 — Ectopic expression of fluorescent BUD-6 fusion constructs did not interfere with colony development. (A) Colony morphology of wild type N150, NECL46-9 and NECL47-5 after 24 and 48 hours of growth on Vogel's medium at 28°C. No differences with respect to general colony architecture or conidiation pattern were observed between wild type and transformants. (B) Colony extension rates were measured every two hours over a period of 24 h and statistically analyzed (C). No significant differences between the three strains could be observed. (TIF) [file pone.0030372.s002.tif]

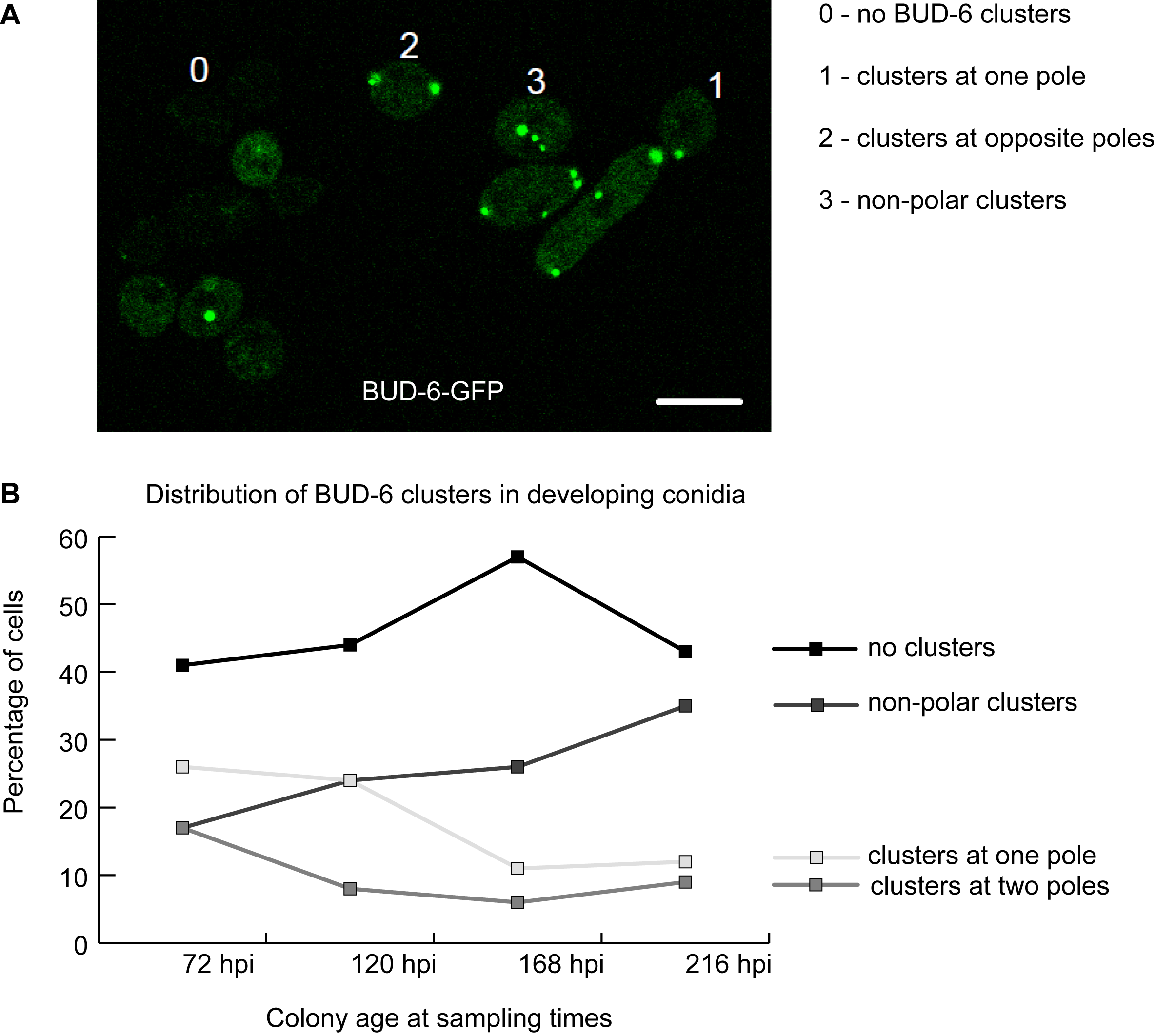

Supplement: Figure S3 — Changes in the abundance of BUD-6 clusters in developing conidia. VMM slants were inoculated with the BUD-6-GFP expressing strain NECL46-9 (Table 2), and continuously incubated for 10 days at 28°C. At the indicated time points conidial samples were taken and observed using laser confocal microscopy. As this analysis was based on sampling only single random optical sections of fields of conidia the results are an underestimate of the number of clusters present (i.e. clusters outside the focal plane were not captured). Each optical section was taken at a random plane through the spores, and thus provides the average distribution pattern within the cell population at each time point. (A) Example image showing the scored pattern of BUD-6 cluster distribution in freshly harvested conidia. Scale bar, 10 µm. (B) The graph displays the percentage of cells showing the particular BUD-6 cluster distribution pattern at the indicated sampling times hours post inoculation (hpi). With colony development the number of conidia with cortical clusters at one or both cell poles decreases, whereas the number of cells containing intracellular clusters increases. Together, this data indicates that the changes in BUD-6 cluster distribution are probably connected to conidial maturation, which causes the redistribution of BUD-6 from sites of cytokinesis to internal compartments. (TIF) [file pone.0030372.s003.tif]
